# IL-33 acts as a costimulatory signal to generate alloreactive Th1 cells in graft-versus-host disease

**DOI:** 10.1172/JCI150927

**Published:** 2022-06-15

**Authors:** Gaelen K. Dwyer, Lisa R. Mathews, José A. Villegas, Anna Lucas, Anne Gonzalez de Peredo, Bruce R. Blazar, Jean-Philippe Girard, Amanda C. Poholek, Sanjiv A. Luther, Warren Shlomchik, Hēth R. Turnquist

**Affiliations:** 1 Department of Immunology, University of Pittsburgh, Pittsburgh, Pennsylvania, USA.; 2 Thomas E. Starzl Transplantation Institute and; 3 Department of Surgery, University of Pittsburgh School of Medicine, Pittsburgh, Pennsylvania, USA.; 4 Department of Biochemistry, University of Lausanne, Epalinges, Switzerland.; 5 Institut de Pharmacologie et de Biologie Structurale, Université de Toulouse, Centre National de la Recherche Scientifique, Université Paul Sabatier, Toulouse, France.; 6 Department of Pediatrics, University of Minnesota Medical School, Minneapolis, Minnesota, USA.; 7 Division of Pediatric Rheumatology, and; 8 Department of Medicine, University of Pittsburgh School of Medicine, Pittsburgh, Pennsylvania, USA.; 9 McGowan Institute for Regenerative Medicine, University of Pittsburgh, Pittsburgh, Pennsylvania, USA.

**Keywords:** Immunology, Transplantation, Bone marrow transplantation, Costimulation, Th1 response

## Abstract

Antigen-presenting cells (APCs) integrate signals emanating from local pathology and program appropriate T cell responses. In allogeneic hematopoietic stem cell transplantation (alloHCT), recipient conditioning releases damage-associated molecular patterns (DAMPs) that generate proinflammatory APCs that secrete IL-12, which is a driver of donor Th1 responses, causing graft-versus-host disease (GVHD). Nevertheless, other mechanisms exist to initiate alloreactive T cell responses, as recipients with disrupted DAMP signaling or lacking IL-12 develop GVHD. We established that tissue damage signals are perceived directly by donor CD4^+^ T cells and promoted T cell expansion and differentiation. Specifically, the fibroblastic reticular cell–derived DAMP IL-33 is increased by recipient conditioning and is critical for the initial activation, proliferation, and differentiation of alloreactive Th1 cells. IL-33 stimulation of CD4^+^ T cells was not required for lymphopenia-induced expansion, however. IL-33 promoted IL-12–independent expression of Tbet and generation of Th1 cells that infiltrated GVHD target tissues. Mechanistically, IL-33 augmented CD4^+^ T cell TCR-associated signaling pathways in response to alloantigen. This enhanced T cell expansion and Th1 polarization, but inhibited the expression of regulatory molecules such as IL-10 and Foxp3. These data establish an unappreciated role for IL-33 as a costimulatory signal for donor Th1 generation after alloHCT.

## Graphical abstract

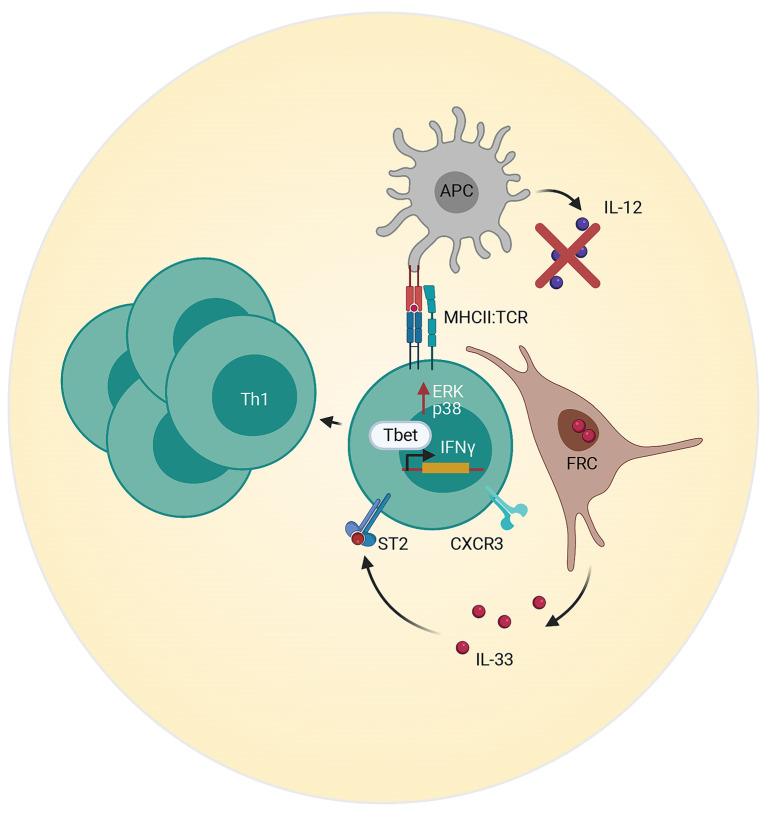

## Introduction

Allogeneic hematopoietic stem cell transplantation (alloHCT) is a curative therapy for high-risk hematopoietic malignancies and is also used to correct life-threatening lymphohematopoietic disorders. alloHCT has also shown promise as a method for inducing tolerance to solid organ transplants. Transplant products include donor T cells to support engraftment and posttransplant immunity and to mediate the removal of malignant cells. Unfortunately, donor T cell responses to polymorphic host MHC molecules and minor histocompatibility (miH) antigens often lead to graft-versus-host disease (GVHD). Despite evolving prophylaxis strategies (1–3), acute GVHD remains a common life-threatening complication that typically arises by 3 to 12 weeks after alloHCT, when alloreactive donor T cells infiltrate and destroy host tissues, particularly the skin, liver, and gastrointestinal (GI) tract (4). GVHD causes significant morbidity and mortality in the treatment of malignancy (4). The risk of GVHD is also a barrier to developing alloHCT for correction of genetic disorders or inducing tolerance to organ transplants (5, 6). Targetable mechanisms to prevent or limit GVHD are of considerable interest.

Alloreactive CD4^+^ Th1 cells and type 1 CD8^+^ T (Tc1) cells producing IFN-γ and TNF-α as well as lytic and apoptosis-inducing proteins cause GVHD target tissue damage (7–9). Thus, methods to limit their generation or functional impact after alloHCT are being explored (10, 11). In general, it is thought that type 1 responses are generated when materials containing pathogen-associated molecular patterns (PAMPs) or damage-associated molecular patterns (DAMPs) are recognized by pattern recognition receptors (PRRs) on antigen-presenting cells (APCs). These stimuli upregulate MHC and costimulatory molecules, but also dictate APC cytokine production to direct the differentiation of interacting T cells into the subsets necessary for effective antipathogen responses (12–14). For example, microbial PAMP recognition by PRRs, particularly the TLRs, initiates APC secretion of IL-12 that activates STAT4 signaling to generate pathogen-clearing Th1 and Tc1 cells (11). Yet alloHCT involves a distinct immunobiology relative to antipathogen responses, as it creates conditions where a high frequency of donor T cells can respond with varied TCR affinity directly or indirectly to pervasive and persistent alloantigens. Furthermore, recipient conditioning with radiation or chemotherapeutic agents to make space for donor cells and prevent their rejection causes lymphodepletion, leading to cytokine-driven homeostatic expansion of donor T cells (15). In addition, myeloablative recipient conditioning causes tissue injury that introduces PAMPs from intestinal microbiota after barrier tissue breakdown and causes ample release of typically sequestered self-derived DAMPs (16). While PAMP activation of recipient APCs has emerged as an important contributor to GVHD pathology, recipients with disrupted PAMP-signaling pathways or lacking recipient APC-expressed costimulatory molecules and IL-12 can still develop GVHD (17). Additionally, GVHD is less severe when donor CD4^+^ T cells lack MyD88 signaling, which favors the survival and differentiation of Th1, Tc1, and Th17 cells (18, 19). These data suggest that recipient-derived DAMPs targeting donor T cells exist and are of substantial importance due to their ability to stimulate donor T cells directly and initiate GVHD.

IL-33 is constitutively expressed and sequestered in the nucleus of cells expressing it, which include epithelial cells and mesenchymal cells of barrier tissues, endothelial cells of blood vessels, and fibroblastic reticular cells (FRCs) in the secondary lymphoid organs (SLOs) (20). Consistent with described DAMP functions for IL-33, it is inactivated by caspase cleavage before apoptotic cell death, but full-length IL-33 is biologically active when released from necrotic cells (20). In the current studies, we have used transgenic mice, allowing precise targeting of donor T cell IL-33 signaling or recipient expression of IL-33 in multiple alloHCT models of CD4^+^ T cell–mediated GVHD, to make what we believe are novel observations into how stromal cell–derived, DAMP-mediated stimulation of donor T cells is critical to pathologic alloimmune responses after alloHCT. Specifically, we identified how IL-33 from FRCs of the SLOs can contribute to the initial activation and proliferation of alloreactive CD4^+^ T cells. This early IL-33 signaling to donor CD4^+^ T cells acts independently of IL-12 to promote Tbet^+^ Th1 differentiation and effector functions. This activity was due to IL-33 acting as a costimulatory signal that augments early TCR-signaling pathways in donor alloreactive CD4^+^ T cells. Our findings identify IL-33 as a highly desirable therapeutic target at the time of alloHCT for preventing acute GVHD.

## Results

### IL-33 functions independently of IL-12 to drive Th1 differentiation and lethal GVHD.

**Figure 1:**
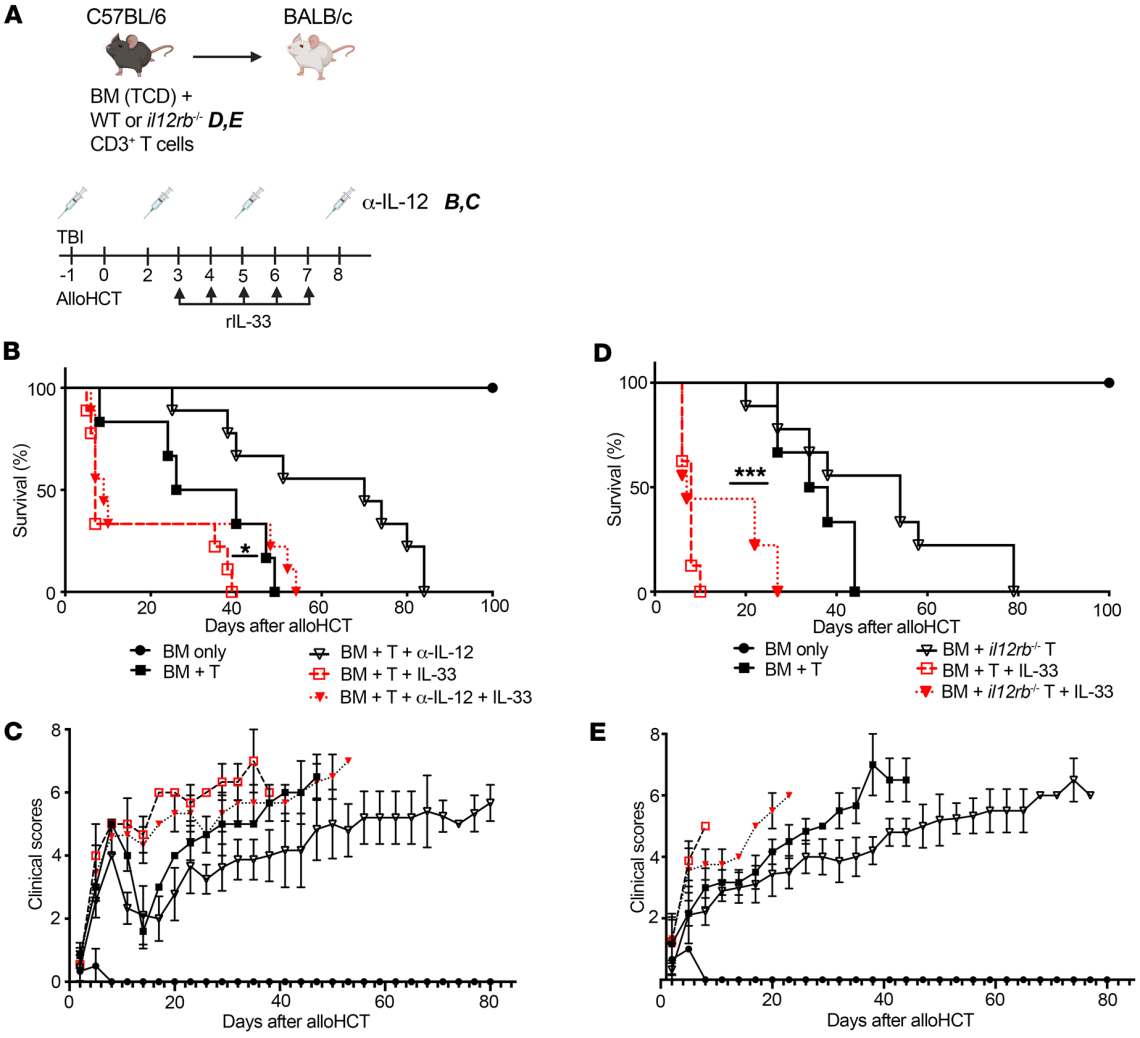
Administration of IL-33 after alloHCT increases the severity of GVHD independent of IL-12. (**A**–**E**) On d–1 before transplant, BALB/c recipient mice received lethal TBI (8 Gy) and cohorts received anti–IL-12p40 (α–IL-12p40) (500 μg/mouse/d) or IgG as control, and this treatment was repeated every 3 days (d2, d5, and d8). On d0, recipients received 1 × 10^7^ WT C57BL/6 (B6) T cell–depleted bone marrow (TCD-BM) alone or with 2 × 10^6^ B6 CD3^+^ T cells (**B**) or 2 × 10^6^ *il12rb2^–/–^* B6 CD3^+^ T cells (**C**) by i.v. injection. Cohorts were treated i.p. with rIL-33 (from d3 to d7 after alloHCT; 0.5 μg/mouse/d) or PBS. (**A**) Model schematic as it relates to IgG or α–IL-12p40 (**B** and **C**) donor T cells (**D** and **E**) and rIL-33 treatments (**B**–**E**). (**B**) Survival graph depicting the influence of rIL-33 with anti–IL-12p40–treated group or (**D**) on *il12rb2^–/–^* CD3^+^ T cells. (**C**) Clinical scores depicting the influence of rIL-33 with α–IL-12p40–treated group or(**E**) on *il12rb2^–/–^* CD3^+^ T cells. Kaplan-Meier survival curve was used for **B** and **D**; **C** and **E** show clinical scores. *n* = 6–9/group. *P < 0.05; ***P < 0.001.

Th1 cells are distinguished by their expression of Tbet, which binds to and activates the gene for IFN-γ (21–23). Tbet also controls the expression and function of CXCR3 and P-selectin that facilitate Th1 cell migration into inflamed tissues (24). The process of Th1 differentiation involves 2 phases of Tbet expression, a first phase stimulated by TCR engagement and IFN-γ, and a second, IL-12–enforced phase (23). IL-12 is a contributor to GVHD, and targeting IL-12p40 alleviates GVHD lethality (11, 25, 26). In vitro, IL-12 promotes Tbet-dependent expression of the IL-33 receptor serum stimulation-2 (ST2) (27) and augments T cell IFN-γ production (28, 29). We have also established that *St2^–/–^* T cells display significantly less IFN-γ expression at day 10 (d10) after alloHCT in fully MHC mismatched recipients (129 to BALB/c; BALB/c to B6; ref. 29). This reduced IFN-γ expression has been associated with decreased GVHD mortality and was consistent with IFN-γ contributing to GVHD lethality (8, 25). To determine whether IL-12 contributed to IL-33–mediated activation and differentiation of donor CD4^+^ T cells after alloHCT in a B6 to BALB/c model, we neutralized IL-12 via anti–IL-12p40 antibodies alone or with IL-33 treatment (Figure 1A). While we confirmed our earlier finding (29) that treatment with rIL-33 after alloHCT accelerates death from GVHD (Figure 1B), we revealed that targeting IL-12p40 after alloHCT did not provide any protection against IL-33–driven GVHD lethality (Figure 1B) or morbidity (Figure 1C). Anti–IL-12p40 delivery alone delayed death, thus confirming effective therapeutic IL-12 blockade. IL-12–independent promotion of GVHD by IL-33 was further verified using *il12rb2^–/–^* donor T cells, which again suggested no role for IL-12 in IL-33–augmented GVHD lethality (Figure 1D) or clinical scores (Figure 1E). These data suggest that the mechanism by which IL-33 drives donor T cell pathology in GVHD is independent of IL-12.

**Figure 2:**
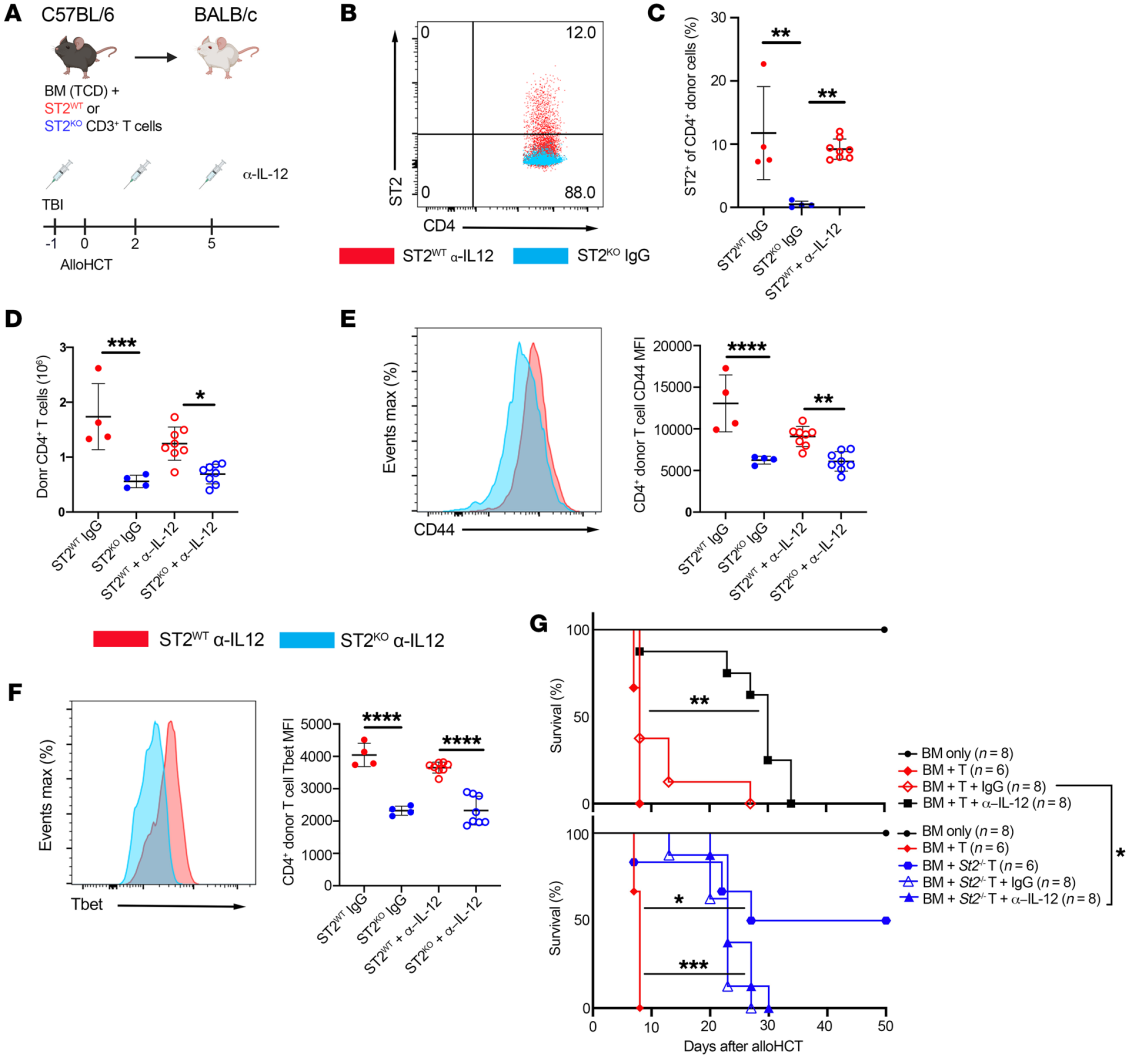
IL-33 stimulation after alloHCT expands ST2^+/+^ CD4^+^ donor T cells and contributes to GVHD lethality independently of IL-12. (**A**–**G**) On d–1, BALB/c recipient mice (**A**–**F**) received α–IL-12p40 or IgG as control (as in Figure 1) and received lethal TBI. On d0, mice received 1 × 10^7^ B6 TCD-BM with 2 × 10^6^ CD90.2^+^ B6 *St2^–/–^* (ST2^KO^) or CD90.1^+^*St2^+/+^* (ST2^WT^) CD3^+^T cells. Total splenocytes were assessed at d7 by flow cytometry. (**A**) Model schematic. (**B**) Representative ST2 expression on donor CD4^+^CD90.1^+^H2-K^d–^ ST2^WT^ (red) and CD4^+^CD90.1^–^H2-K^d–^ ST2^KO^ (blue) cells from recipient’s spleens at d7. (**C**) Frequency of ST2^+^ donor CD90.1^–^ST2^KO^ and CD90.1^+^ST2^WT^ CD4^+^ T cells. (**D**) Donor CD90.1^–^ST2^KO^ and CD90.1^+^ST2^WT^ CD4^+^ T cells counts in recipients that received α–IL-12p40 or IgG. (**E**) Representative CD44 expression on CD90.1^–^ST2^KO^ (blue) and CD90.1^+^ST2^WT^ (red) CD4^+^ T cells and quantification of CD44 MFI. (**F**) Representative Tbet expression on CD90.1^–^ST2^KO^ (blue) and CD90.1^+^ST2^WT^ (red) CD4^+^ T cells and quantification of Tbet MFI. (**G**) Survival graph depicting the influence of donor T cell ST2 deletion alone or with IL-12 neutralization on GVHD lethality. Data in **B**–**F** are represented as mean ± SD. Kaplan-Meier survival curve was used for **G**. *n* = 6–8/group. *P < 0.05; **P < 0.01; ***P < 0.001; ****P < 0.0001, 1-way ANOVA (**C**–**F**).

To further define the impact of neutralizing IL-12 on IL-33–mediated early donor CD4^+^ T cell activation and differentiation, we performed alloHCT experiments in which donor B6 *St2^–/–^* and *St2^+/+^* T cells were adoptively transferred into distinct (Figure 2, A–F) or the same (Supplemental Figure 1, A–E; supplemental material available online with this article; https://doi.org/10.1172/JCI150927DS1) BALB/c recipients with IL-12p40 blockade. Unlike in in vitro studies in which IL-12 increased T cell ST2 expression (28, 29), neutralization of IL-12p40 did not significantly modulate the frequency of donor ST2^+^CD4^+^ cells at d7 after alloHCT (Figure 2, B and C). IL-33 stimulation of donor CD4^+^ T cells was crucial for their expansion in the presence or absence of IL-12p40 stimulation given that CD4^+^ T cells were decreased significantly when they lacked the IL-33 receptor ST2 regardless of whether anti–IL-12p40 or IgG was delivered (Figure 2D). These differences were not due to differences in alloimmune-mediated inflammation between recipients of *St2^–/–^* or *St2^+/+^* T cells, as *St2^–/–^* T cells cotransferred with *St2^+/+^* T cells into the same recipient failed to expand at the same rate as WT cells (Supplemental Figure 1, A–C). IL-12 neutralization was not as impactful as the lack of IL-33 stimulation on donor CD4^+^ T cell activation or Th1 differentiation based on CD44 (Figure 2E) and Tbet expression (Figure 2F). *St2^+/+^* T cells exhibited significantly higher MFI for CD44 and Tbet than *St2^–/–^* T cells, regardless of IL-12p40 blockade. These findings were recapitulated when *St2^–/–^* and *St2^+/+^* T cells were adoptively transferred into the same recipient (Supplemental Figure 1, D and E). *St2^–/–^* T donor cells caused less lethal GVHD than did WT T cells, with or without IL-12 antagonism (Figure 2G). These data support that ST2 signaling is critical for early T cell activation, expansion, and differentiation through a mechanism independent of IL-12.

### AlloHCT conditioning upregulates IL-33 protein expression in the T cell zones of the spleen.

**Figure 3:**
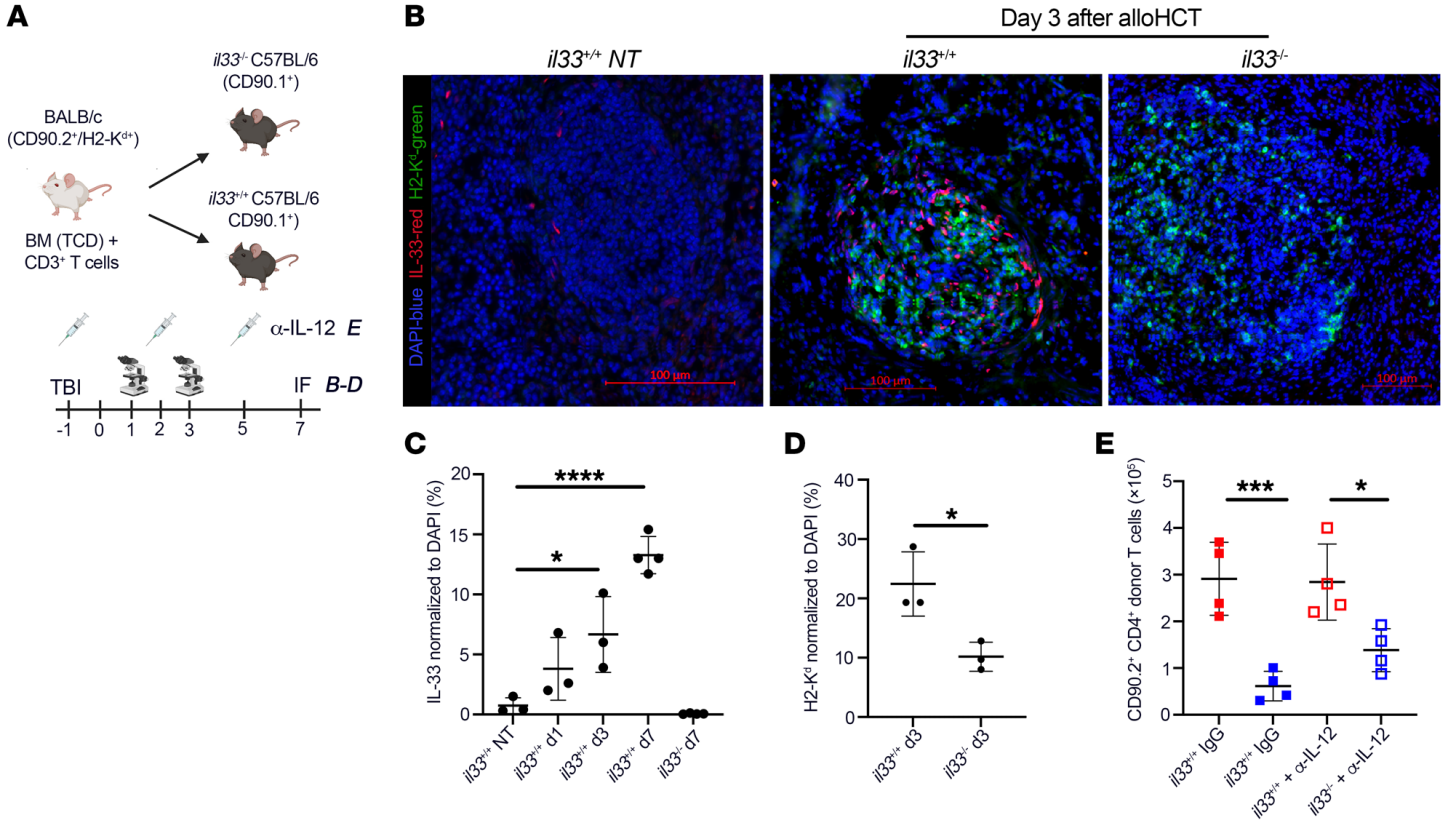
alloHCT conditioning increases recipient IL-33 expression in the spleen and is necessary for donor T cell expansion independently of IL-12. (**A**–**E**) On d–1, CD90.1^+^*il33^–/–^* or CD90.1^+^*il33^+/+^* B6 recipient mice received lethal TBI (11 Gy), and some cohorts received anti–IL-12p40 or IgG as control (as described in Figure 1). On d0, mice received 1 × 10^7^ H2-K^d+^CD90.2^+^ BALB/c TCD-BM with 2 × 10^6^ H2-K^d+^CD90.2^+^ BALB/c CD3^+^T cells. Donor H2-K^d+^ splenocytes were assessed at d1, d3, and d7 by immunofluorescence microscopy and at d7 by flow cytometry. (**A**) Model schematic as it relates to imaging (**B**–**D**) and antibody (IgG or anti–IL-12p40) treatments (**E**). (**B**) Representative spleen image from each cohort on d3. (**C**) Frequency of IL-33^+^ cells in the recipient and naive spleens; 1 complete scanned cross section was analyzed per mouse. (**D**) Frequency of H2-K^d+^ donor T cells in recipient spleens at d3, analyzed from 1 complete scanned cross section per mouse. (**E**) Donor CD90.2^+^CD4^+^ cell counts from the spleen on d7. Data in **B**–**F** are represented as mean ± SD. *n* = 3–4/group. *P < 0.05; ***P < 0.001; ****P < 0.0001, 1-way ANOVA (**B** and **E**); Student’s *t* test (**D**).

We previously reported that IL-33 is upregulated in the small intestine (SI) following total body irradiation (TBI) and during GVHD (29). We observed a similar upregulation of IL-33 in the SLOs (Figure 3, A–D). IL-33 expression was increased 5- to 6-fold by d3 after alloHCT and 12- to 13-fold by d7 after alloHCT in the spleen of lethally irradiated B6 *il33^+/+^* recipients compared with *il33^–/–^* recipients (Figure 3, B and C). IL-33 was primarily expressed in the T cell zone of the spleen, which is where CD4^+^ donor T cells home and proliferate at d3 (30, 31). Consistent with a role for IL-33 in CD4^+^ T cell expansion, we also observed donor H2-K^d+^ T cells adjacent to IL-33–expressing cells, with reduced frequency of donor H2-K^d+^ T cells in *il33^–/–^* recipients compared with *il33^+/+^* recipient spleens (Figure 3, B and D). Thus, IL-33 is upregulated following alloHCT conditioning in the SLO in areas of high donor T cell trafficking, proliferation, and differentiation.

We next utilized B6 CD90.1^+^*il33^+/+^* and *il33^–/–^* mice to assess the impact of neutralizing IL-12 in the absence of recipient IL-33 on WT donor T cell expansion. In this fully MHC-mismatched model in which CD90.1^+^*il33^+/+^* and *il33^–/–^* recipients received WT CD90.2^+^ BALB/c donor BM and T cells (Figure 3A), we observed that donor BALB/c (CD90.2^+^) CD4^+^ T cells isolated from the spleen of *il33^–/–^* recipients did not expand at the same rate as donor CD4^+^ T cells isolated from *il33^+/+^* recipients on d7 after alloHCT (Figure 3E). This decreased expansion was independent of IL-12, since donor CD90.2^+^CD4^+^ T cells isolated from CD90.1^+^*il33^+/+^* recipients that received IgG had expansion similar to that of T cells isolated from recipients that received IL-12 blockade (Figure 3E). These findings support the overall concept that a dominant role of recipient IL-33 after alloHCT is to directly promote CD4^+^ T cell proliferation independently of IL-12 signaling. These data also point to a relationship in which CD4^+^ T cells, in addition to APCs, sense and respond to stimulatory DAMP signals released from nonhematopoietic recipient cells.

### IL-33 augmented in FRCs supports alloreactive CD4^+^ T cells.

**Figure 4:**
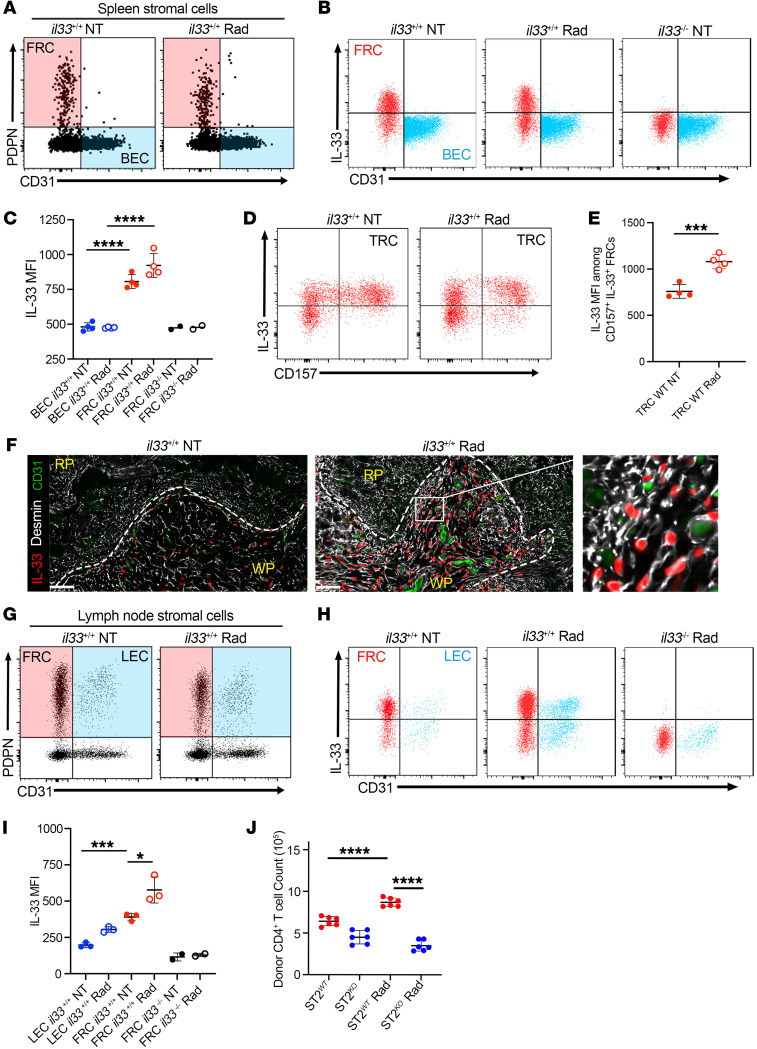
FRCs increase IL-33 after irradiation to stimulate ST2-dependent donor CD4^+^ T cell expansion. (**A**–**I**) WT B6 mice received lethal TBI (11 Gy; Rad [radiation treated]) on d0. On d1, spleen and LN samples were compared with those from nonirradiated WT and *il33^–/–^* B6 mice. (**A**–**E**) Flow cytometric analysis of splenic stromal cell isolates. (**A**) Representative dot plots of live TER119^–^CD45^–^ pregated stromal cells highlighting PDPN^+^CD31^–^FRCs (red) and CD31^+^PDPN^–^ blood endothelial cells (BECs) (blue). (**B**) IL-33 expression by FRCs and BECs. *il33^–/–^* B6 mice served as a negative control. (**C**) MFI for IL-33 in the IL33^+^ gate of BECs and FRCs. (**D**) Representative plots showing IL-33 expression in PDPN^+^FRC subsets using CD157 to identify TRCs. (**E**) IL-33 MFI in the IL33^+^ gate of splenic TRC. (**F**) Spleen staining for IL-33 (red) and fibroblast (desmin; white) and vascular (CD31; green) markers. The magnified region highlights IL-33 within desmin^+^ white pulp FRCs. Dotted lines depict red pulp (RP) and white pulp (WP) borders. Scale bars: 50 μm. (**G**) Representative plots of LN stromal isolates gated as in **A** and highlighting PDPN^+^CD31^–^FRCs (red) versus CD31^+^PDPN^+^ lymphatic endothelial cells (LECs) (blue). (**H**) IL-33 expression in FRCs versus LECs. (**I**) MFI for IL-33 in the IL33^+^ gate of FRCs and LECs. (**J**) Purified B6 LN FRCs were either irradiated (3.5 Gy) or left untreated, and after 24 hours, FRCs were cultured with TCD and irradiated (3.5 Gy) B6 splenocytes and BALB/c *St2^+/+^* or *St2^–/–^* T cells. Flow cytometry was used to quantify CD4^+^ T cells on d3. Data are represented as mean ± SD. *P < 0.05; ***P < 0.001; ****P < 0.0001, 1-way ANOVA. Data are representative of at least 2 experiments.

To precisely identify the source of IL-33 in recipient SLOs following conditioning, we assessed IL-33 expression in the spleen and the LNs on d1 following TBI (Figure 4, A–I). Nonhematopoietic (CD45^–^) podoplanin^+^ (PDPN^+^) CD31^–^ FRCs were the dominant source of IL-33 both before and following irradiation. As suggested by recent scRNA-Seq data (32), CD157^+^ T cell zone reticular cells (TRCs) express IL-33 (Figure 4D) and display a profound increase in IL-33 after TBI (Figure 4E). This was consistent with increased IL-33 in desmin^+^ fibroblasts of the central white pulp (Figure 4F). FRCs are the dominant source of IL-33 in LN stroma before and after irradiation, with lymphatic endothelial cells contributing to a lesser extent (Figure 4, G–I). FRCs express the T cell–attracting chemokines CCL19 and CCL21 (33) and are ideally suited to delivering an IL-33 signal to attracted alloreactive donor T cells. To determine whether FRCs act as a source of IL-33–stimulating donor CD4^+^ T cells, we isolated CD45^–^CD31^–^PDPN^+^ FRCs and added them with or without prior irradiation to a mixed leukocyte reaction (MLR) between BALB/c T cells and T cell–depleted, irradiated B6 splenocytes. Consistent with our in vivo observations, IL-33 from irradiated FRCs augmented CD4^+^ T cells in an ST2-dependent fashion (Figure 4J). These data demonstrate that upregulated IL-33 in FRCs is a functionally important source of IL-33 for donor CD4^+^ T cell stimulation.

### IL-33 stimulation augments early T cell expansion following an alloantigen encounter.

**Figure 5:**
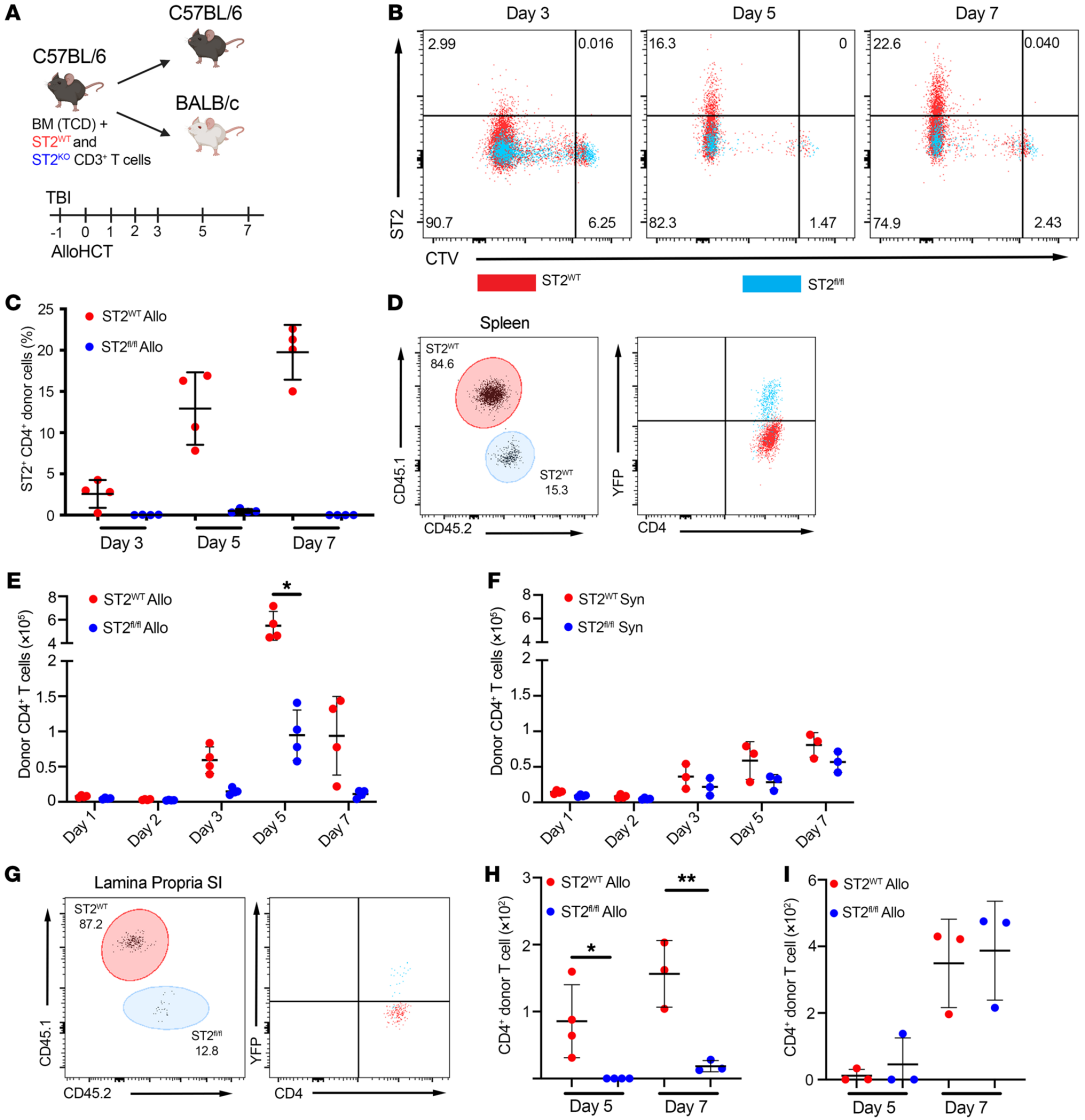
Early donor CD4^+^ T cell expansion is dependent on IL-33. (**A**–**H**) BALB/c (allo) and B6 (syn) recipient mice received lethal TBI (8 Gy or 11 Gy, respectively) on d–1. On d0, the recipient mice received 1 × 10^7^ WT B6 TCD-BM with 1 × 10^6^ CD3^+^ T cells from CD45.2^+^ *CD4-Cre*×*R26-LSL-YFP*×*St2^fl/fl^* (ST2*^fl/fl^*) and 1 × 10^6^ CD3^+^ T cells from *St2^+/+^* CD45.1^+^ (ST2^WT^) B6 mice. T cells were labeled with CTV prior to adoptive transfer. T cells were harvested from the spleen on d1, d2, d3, d5, and d7 after alloHCT and from the SI lamina propria on d5 and d7 and assessed by flow cytometry. (**A**) Model schematic. (**B**) Representative plots of ST2 expression on donor CD4^+^CD45.1^+^H2-K^d–^ ST2^WT^ (red, quadrant frequencies) and CD4^+^CD45.2^+^H2-K^d–^YFP^+^ ST2*^fl/fl^* (blue) cells isolated from the spleen of the same allo recipient. (**C**) Frequency of ST2 on donor CD45.1^+^ or CD45.2^+^CTV^lo^ donor T cells on d3, d5, and d7. (**D**–**F**) Representative plot of CD45.1^+^YFP^–^ and CD45.2^+^YFP^+^ donor CD4^+^ T cells of an allo recipient (**D**) and total ST2^WT^ (red) versus ST2*^fl/fl^* (blue) donor CD4^+^ T cells from the spleen of (**E**) allo or (**F**) syn recipients on the indicated day. (**G**) Representative plot of CD4^+^ donor ST2^WT^ (red) versus ST2*^fl/fl^* (blue) from the SI lamina propria of an allo recipient. (**H**–**I**) total ST2^WT^ (red) versus ST2*^fl/fl^* (blue) donor CD4^+^ T cell counts from the SI (**H**) allo recipients or (**I**) syn recipients. Data in **A**–**I** are represented as mean ± SD. *n* = 3–4/group. Data are representative of 2 independent experiments. *P < 0.05; **P < 0.01, 2-way ANOVA (**C**–**I**).

The ST2/IL-33 axis is broadly implicated in organ and tissue homeostasis as well as diverse immune cell development and functions (34). To rule out any indirect defects due to the global lack of IL-33 or ST2, we generated B6 CD45.2^+^ *CD4-Cre*×*ROSA(R)26-LoxP-Stop-LoxP(LSL)-YFP*×*St2^fl/fl^* mice. Using these mice allowed us to focus on YFP^+^ST2^–^CD4^+^ T cells in CD4-driven GVHD models (35). We first sought to determine whether IL-33 was acting directly on donor CD4^+^ T cells early during activation in the SLOs to cause the striking decrease in numbers of *St2^–/–^* donor CD4^+^ T cells (Figure 2D and Supplemental Figure 1C) and decreased CD4^+^ T cell numbers in *il33^–/–^* recipients (Figure 3E) on d7 after alloHCT. Donor CD3^+^ T cells from CD45.2^+^*CD4-Cre*×*R26-LSL-YFP*×*St2^fl/fl^* (ST2*^fl/fl^*) and CD45.1^+^*St2^+/+^* (ST2^WT^) mice were labeled with CellTrace Violet (CTV) and cotransferred into the same lethally irradiated BALB/c (allogeneic [allo]) or B6 (syngeneic [syn]) recipients (Figure 5A). CD4^+^ T cells were harvested from the spleen on d1, d2, d3, d5, and d7 to determine the effect of IL-33 stimulation on early expansion after alloHCT (Figure 5A). ST2*^fl/fl^* CD4^+^ T cells were identified as a YFP^+^ subset to ensure they had expressed the Cre recombinase and the absence of ST2 on YFP^+^ cells was verified (Figure 5, B–C and G, and Supplemental Figure 2A). ST2 was upregulated on donor ST2^WT^ CD4^+^ T cells from both allo and syn recipients (Figure 5, B and C, and Supplemental Figure 3, A and B), but more rapidly and strongly in allo recipients (Figure 5B and Supplemental Figure 3A). The frequency of donor ST2^+^CD4^+^ cells steadily increased from d3 to d7 in the ST2^WT^ T cell subset isolated from allo BALB/c recipients (Figure 5, B and C). BALB/c recipients exhibited a reduced number of presumed alloantigen-specific ST2*^fl/fl^* CD4^+^ T cells compared with ST2^WT^ CD4^+^ T cells in the spleen of the same recipient on d3, d5, and d7 (Figure 5, D and E). Interestingly, IL-33 signaling was not necessary for lymphopenia-induced proliferation (LIP) in the spleen of syn recipients, as we observed a similar number of ST2*^fl/fl^* and ST2^WT^ donor CD4^+^ T cells (Figure 5F). Additionally, a lack of early IL-33 stimulation presumably in the SLO resulted in a failure of donor ST2*^fl/fl^* CD4^+^ T cells to infiltrate GVHD target tissues in allo recipients (Figure 5, G and H). This was not the case in the syn recipients, in which both the ST2*^fl/fl^* CD4^+^ T cells and the ST2^WT^ CD4^+^ T cells successfully infiltrated the SI by d7 (Figure 5I). Thus, IL-33 does not appear to function like IL-7, which augments weak TCR signaling to self-peptides presented on self-MHC to support LIP after irradiation (36). Instead, IL-33 predominantly promotes the expansion of alloreactive CD4^+^ T cells, suggesting that IL-33 augments the expansion of CD4^+^ T cells following TCR recognition of allo MHC or miH.

### Early T cell activation is enhanced by IL-33.

**Figure 6:**
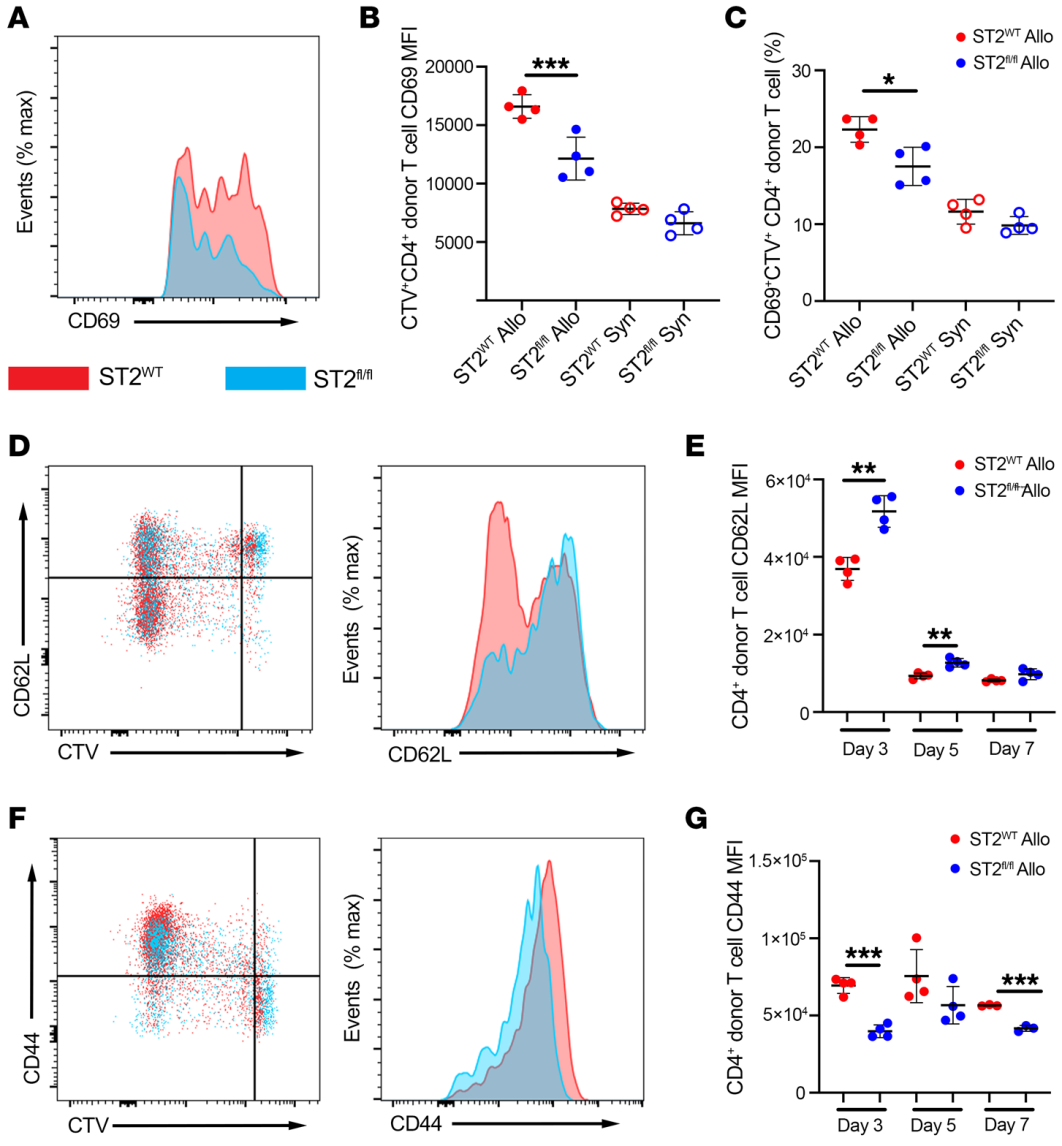
IL-33 stimulation augments early donor CD4^+^ T cell activation. (**A**–**G**) CD3^+^ T cells from CD45.2^+^ *CD4-Cre*×*R26-LSL-YFP*×*St2^fl/fl^* (ST2*^fl/fl^*) B6 (1 × 10^6^) and *St2^+/+^* CD45.1^+^ (ST2^WT^) B6 (1 × 10^6^) mice were labeled with CTV and cotransferred with 1 × 10^7^ WT B6 TCD-BM into lethally irradiated BALB/c and B6 recipients. T cells were isolated from the spleens on d1, d2, d3, d5, and d7 and assessed by flow cytometry. (**A**) Representative CD69 expression on ST2^WT^ (red) CD45.1^+^CTV^+^ or ST2*^fl/fl^* (blue) CD45.2^+^CTV^+^ donor CD4^+^ T cells from the same allo recipient spleen on d1. (**B**) Quantification of CD69 MFI on CD45.1^+^ or CD45.2^+^ CTV^+^ donor CD4^+^ T cells from allo recipient spleens on d1. (**C**) Quantification of frequency of CD45.1^+^ or CD45.2^+^CTV^+^ donor CD69^+^ CD4T cells at d1. (**D**) Representative plot (CTV versus CD62L) and histogram of CD62L expression on ST2^WT^ (red) or ST2*^fl/fl^* (blue) donor CD4^+^ T cells from an allo recipient spleen on d3. (**E**) Quantification of CD62L MFI on d3, d5, and d7. (**F**) Representative plot (CTV versus CD44) and histogram of CD44 expression on ST2^WT^ (red) or ST2*^fl/fl^* (blue) donor CD4^+^ T cells from an allo recipient spleen on d3. (**G**) Quantification of CD44 MFI on d3, d5, and d7. Data shown in **B**–**G** are represented as mean ± SD. *n* = 3–4/group. Data are representative of 2 experiments. *P < 0.05; **P < 0.01; ***P <0.001, 1-way ANOVA (**B** and **C**); 2-way ANOVA (**E** and **G**).

Another sensitive marker of TCR engagement is the upregulation of CD69, which can be detected by 2 to 3 hours after TCR engagement by a non-self-antigen (37, 38). As early as d1 after alloHCT, ST2^WT^ CD4^+^ T cells have a higher CD69 MFI and an increased frequency of ST2^WT^ CD4^+^ donor T cells expressing CD69 relative to ST2*^fl/fl^* cells from the same allo recipient spleens (Figure 6, A–C). There was no appreciable difference in CD69 MFI or frequency of donor CD69^+^CD4^+^ T cells in the spleens of TBI-conditioned syn recipients (Figure 6, B and C). These data support the conclusion that IL-33 is acting to enhance the activation of alloreactive T cells and are strengthened by our assessment of the total proteome of anti-CD3, anti-CD28, and IL-33 in vitro–stimulated CD4^+^ T cells. Two hours of stimulation with anti-CD3 and anti-CD28 along with IL-33 resulted in a significant upregulation of CD69 protein compared with stimulation with anti-CD3 and anti-CD28 alone (Supplemental Figure 2B). In addition to these noticeable differences in early activation marker CD69, ST2*^fl/fl^* CD4^+^ T cells retained their expression of CD62L through d5 above expression levels of ST2^WT^ CD4^+^ T cells from allo recipients (Figure 6, D and E). ST2^WT^ CD4^+^ donor T cells also expressed higher levels of the activation marker CD44 compared with ST2*^fl/fl^* CD4^+^ T cells isolated from the same allo recipient (Figure 6, F and G). These results indicate that IL-33 is acting to enhance the early activation of alloreactive donor CD4^+^ T cells.

### IL-33 drives Th1 differentiation while inhibiting regulatory gene expression in CD4^+^ T cells after alloHCT.

**Figure 7:**
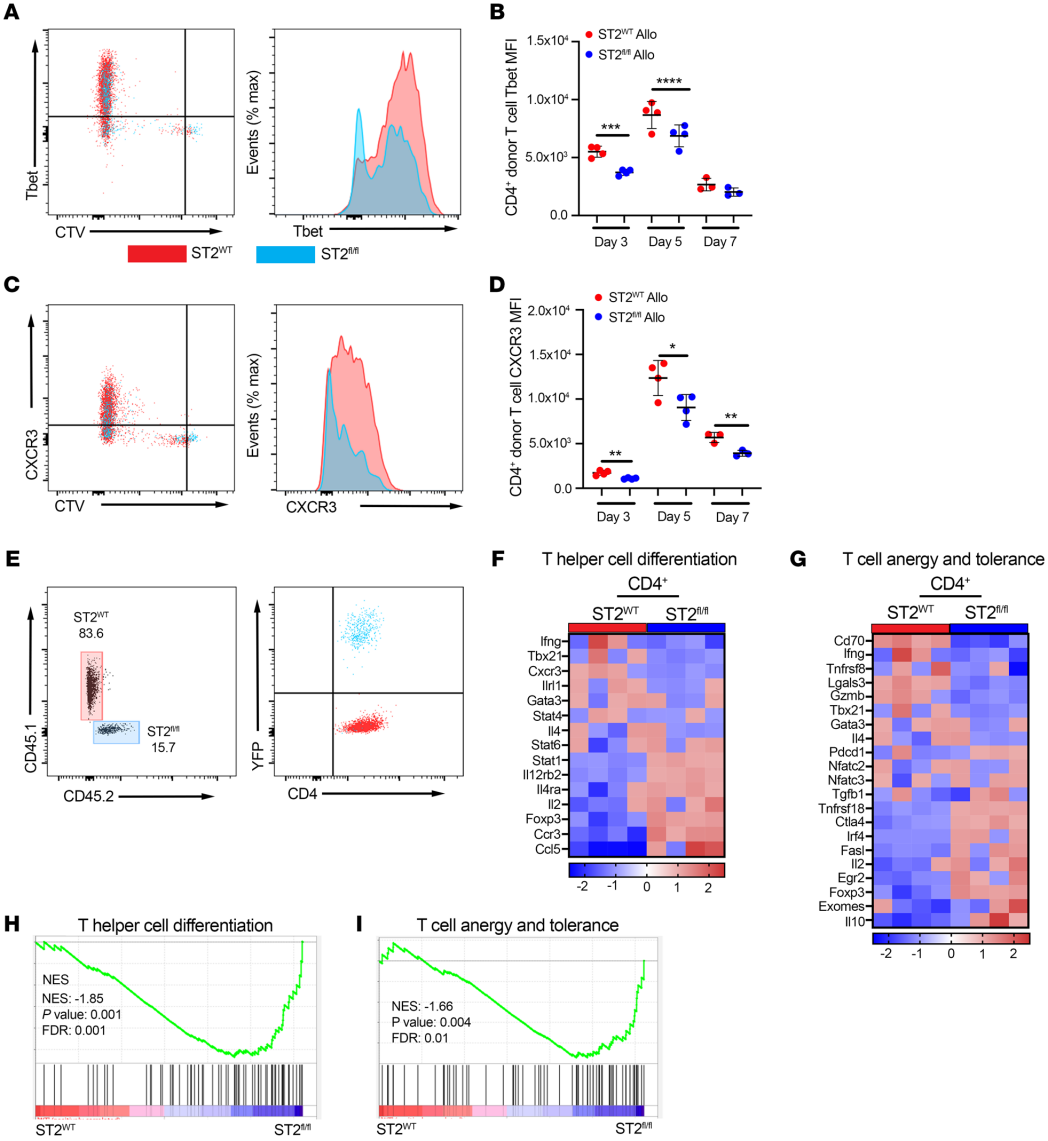
IL-33 stimulation of donor CD4^+^ T cells drives Th1 differentiation while inhibiting regulatory gene expression. (**A**–**D**) CTV-labeled CD3^+^ T cells from CD45.2^+^ *CD4-Cre*×*R26-LSL-YFP*×*St2^fl/fl^* (ST2*^fl/fl^*) B6 (1 × 10^6^) and *St2^+/+^* CD45.1^+^ (ST2^WT^) B6 (1 × 10^6^) mice were cotransferred with 1 × 10^7^ WT B6 TCD-BM into irradiated BALB/c and B6 recipients. Splenocytes were harvested on d1, d2, d3, d5, and d7 for flow analysis. (**A**) Representative plot (CTV versus Tbet) and histogram of Tbet expression on ST2^WT^ (red) or ST2*^fl/fl^* (blue) donor CD4^+^ T cells from the same allo spleen (d5). (**B**) Quantification of Tbet MFI on d3, d5, and d7. (**C**) Representative plot (CTV versus CXCR3) and histogram of CXCR3 expression on ST2^WT^ (red) or ST2*^fl/fl^* (blue) donor CD4^+^ T cells from the same allo spleen (d7). (**D**) Quantification of CXCR3 MFI on d3, d5, and d7. (**E**–**I**) CTV-labeled CD3^+^ T cells from ST2*^fl/fl^* and ST2^WT^ B6 mice were cotransferred into irradiated BALB/c as described in **A**–**D**. CD4^+^ T cells were sorted from the same spleen on d5 for *St2^+/+^* H2-K^d–^CD45.1^+^YFP^–^ (ST2^WT^) and *St2^fl/fl^* H2-K^d–^CD45.2^+^YFP^+^ (ST2*^fl/fl^*) directly into cDNA prep cell lysis buffer. (**E**) Representative sort plot of CD4^+^CD45.1^+^YFP^–^ and CD4^+^CD45.2^+^YFP^+^ donor cells. (**F** and **G**) Heatmaps of T helper cell differentiation and T cell anergy and tolerance-associated genes enriched in ST2^WT^ (red) and ST2*^fl/fl^* (blue) donor CD4^+^ T cells. (**H** and **I**) Leading edge plots of GSEA of ST2^WT^ (red) or ST2*^fl/fl^* (blue) donor CD4^+^ T cells compared with transcriptional profiles of T helper cell differentiation and T cell anergy and tolerance. Data in **A**–**D** are represented as mean ± SD with *n* = 3–4/group. Data are representative of 2 independent experiments. Data in **E**–**I** show *n* = 4/group. *P < 0.05; **P < 0.01; ***P < 0.001; ****P < 0.0001, 2-way ANOVA (**B** and **D**).

Further comparison of donor T cells in the SLO at early time points of T cell activation established that ST2^WT^ CD4^+^ T cells expressed more Tbet than ST2*^fl/fl^* CD4^+^ T cells from the same spleen of allo recipients through d5 after alloHCT (Figure 7, A and B). Tbet induces IFN-γ expression and is critical for upregulation of CXCR3, which controls the alloreactive T cell trafficking to GVHD target tissues (39). CXCR3 was expressed at higher levels on donor ST2^WT^ CD4^+^ T cells than ST2*^fl/fl^* T cells in the same spleen of allo recipients through d7 after alloHCT (Figure 7, C and D). This decrease in ST2*^fl/fl^* T cell CXCR3 matched our earlier finding of decreased infiltration of ST2*^fl/fl^* CD4^+^ T cells into the SI. Our data establish that alloreactive ST2^WT^ T cells are more proliferative, more highly activated, and prone to differentiate into Th1 cells.

To better understand the pathways IL-33 controls after alloHCT in allo recipients, we performed RNA-Seq on sorted splenic ST2*^fl/fl^* and ST2^WT^ donor CD4^+^ T cells cotransferred into the same recipient on d5 after alloHCT (Figure 7E). As anticipated from the above studies, the gene profile of the ST2^WT^ donor CD4^+^ T cells from our RNA-Seq analysis indicated that IL-33 promotes metabolic activity and Myc activation (Supplemental Figure 4, A and B) needed for robust T cell proliferation following TCR stimulation with cognate antigen (40). Interestingly, the ST2*^fl/fl^* donor CD4^+^ T cells from the same allo recipients, represented by the negative normalized enrichment scores (NES) on the left side of curves, were enriched for gene set enrichment analysis (GSEA) pathways for genes expressed in T helper cell differentiation and T cell anergy and tolerance (Figure 7, F–I, and Supplemental Table 1). The heatmap of genes representative of T cell anergy and tolerance and T helper cell differentiation shows that ST2^WT^ donor CD4^+^ T cells expressed higher levels of Th1 genes, including *Ifng*, *Tbx21*, *Cxcr3*, and IFN-induced *Cd70* (Figure 7, F and G). ST2*^fl/fl^* donor CD4^+^ T cells instead had enriched expression of several regulatory genes, including *Foxp3*, *Ctla4*, and *Il10* (Figure 7, F and G), which caused the GSEA results for the T helper differentiation pathway and T cell anergy and tolerance to be enriched in ST2*^fl/fl^* donor CD4^+^ T cells (Figure 7, H and I). These RNA-Seq findings reflected serum cytokine data in GVHD studies in which *il33^–/–^* B6 recipients of WT BALB/c T cells displayed reduced IFN-γ compared with WT recipients (Supplemental Figure 4, C and D) and increased IL-10 for *il33^–/–^* recipients compared with the *il33^+/+^* recipients (Supplemental Figure 4E). The increase in IL-10 was mostly reversed by treating the *il33^–/–^* recipients with rIL-33 from d3 to d6 after alloHCT (Supplemental Figure 4, C and E). These findings imply that IL-33 not only supports Th1 differentiation, but also limits regulatory gene expression by alloreactive donor CD4^+^ T cells.

### Alloantigen TCR activation is augmented by IL-33 stimulation.

Successful CD4^+^ T cell activation and differentiation require an initial signal generated through a robust TCR interaction with cognate antigen/MHC and a second, antigen-independent costimulatory signal (41). While Th1 differentiation is typically thought to be dominated by innate cell-derived cytokine IL-12, the strength of TCR and costimulation signaling are highly influential in dictating CD4^+^ T cells’ differentiation fates (42–44). Strong TCR signals support Th1 generation and limit Foxp3 expression (45–47), whereas an attenuated TCR signal results in CD4^+^ T cell Foxp3 expression (48). Costimulatory molecules feed into TCR-signaling pathways to limit Foxp3 expression in newly activated CD4^+^ T cells (49, 50). Our RNA-Seq data revealed that IL-33 stimulation of CD4^+^ donor T cells augmented expression of *Ifng* and *Cd70* while limiting the expression of *Foxp3* and *Il10* (Figure 7G). These data suggest that IL-33 may be acting as a costimulatory stimulus that amplifies alloreactive TCR signals after alloHCT.

**Figure 8:**
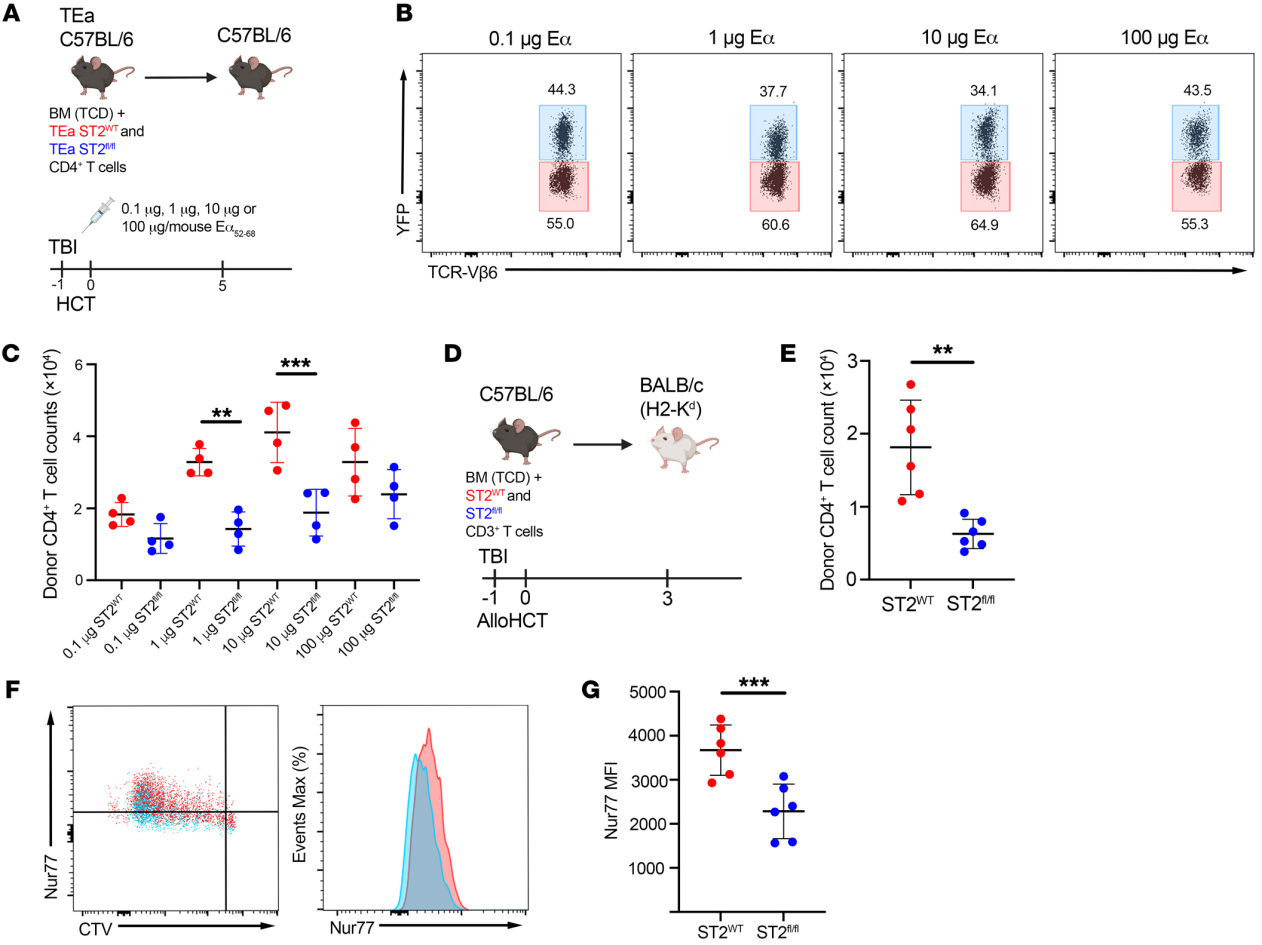
IL-33 boosts TCR signaling to support alloreactive CD4^+^ T cell expansion. (**A**–**C**) B6 mice received lethal TBI (11 Gy) on d–1 and 1 × 10^7^ WT B6 TCD-BM with 1 × 10^6^ *TEa* and 1 × 10^6^ *CD4-Cre*×*R26-LSL-YFP*×*St2^fl/fl^* *TEa* B6 CD4^+^ T cells on d0. They also received the indicated doses of Eα_52–68_ peptide i.p. Splenic T cells were assessed on d5 by flow cytometry. (**A**) Model schematic. (**B**) Representative plots of ST2^WT^ (TCR-Vβ6^+^YFP^–^; red) and ST2*^fl/fl^* *TEa* (TCR-Vβ6^+^YFP^+^; blue) CD4^+^ T cells and (**C**) quantification of their numbers on d5. (**D**–**G**) CTV-labeled CD3^+^ T cells from CD45.2^+^ *CD4-Cre*×*R26-LSL-YFP*×*St2^fl/fl^* B6 (1 × 10^6^) and *St2^+/+^* CD45.1^+^ B6 (1 × 10^6^) mice were cotransferred with 1 × 10^7^ WT B6 TCD-BM into lethally irradiated BALB/c recipients. ST2^WT^ (red) and ST2*^fl/fl^* (blue) CD4^+^ T cells from the same spleen were assessed by flow cytometry on d3. (**D**) Model schematic. (**E**) Comparison of donor ST2^WT^ (red) and ST2*^fl/fl^* (blue) CD4^+^ T cells counts from the same spleen on d3. (**F**) Representative plots and histograms of Nur77 expression. (**G**) Nur77 MFI on d3 from ST2^WT^ (red) and ST2*^fl/fl^* (blue) donor CD4^+^ T cells. Data in **B** and **C** are represented as mean ± SD with *n* = 4/group. Data in **E**–**G** are represented as mean ± SD with *n* = 4–6/group. Data are representative of 2 independent experiments. **P < 0.01; ***P < 0.001, 1-way ANOVA (**C**); paired Student’s *t* test (**E**–**G**).

To precisely show whether IL-33 heightens alloreactive TCR stimulation, we crossed B6 *CD4-Cre*×*R26-LSL-YFP*×*St2^fl/fl^* mice to B6 *TEa* mice, which have CD4^+^ T cells expressing a transgenic TCR recognizing a BALB/c allopeptide (Eα_52–68_) presented on I-A^b^ (51). We cotransferred donor ST2^WT^ and ST2*^fl/fl^* *TEa* CD4^+^ T cells into the same B6 recipient and compared their expansion to increasing doses of Eα_52-68_ peptide delivered with autologous HCT. ST2^WT^*TEa* CD4^+^ T cells (Vβ6^+^YFP^–^) exhibited a dose-dependent escalation in T cell expansion to Eα peptide that was lost in the ST2*^fl/fl^TEa* CD4^+^ (Vβ6^+^YFP^+^) population (Figure 8, A–C). Synergism between allopeptide/I-A^b^/TCR and IL-33 was most notable at low peptide doses and was less apparent at the highest dose (Figure 8C). These data demonstrate that IL-33 can function as a costimulatory signal that synergizes with the TCR to qualitatively increase T cell expansion.

Our data looking at early activation time points also point to IL-33–mediated augmentation of alloantigen-driven TCR signaling, as we saw that IL-33 stimulation augmented the frequency of CD69^+^CD4^+^ donor T cells and the expression of CD69 on B6 ST2^WT^ donor T cells (Figure 6, A–C). While CD69 expression is representative of TCR signaling, CD69 expression can be influenced by inflammatory stimuli (52). Nur77 is an immediate-early response gene expressed in T cells following TCR stimulation (53, 54) and is not induced unless the TCR has been engaged (52). We investigated the impact of IL-33 stimulation on TCR signaling by quantifying Nur77 expression after cotransferring ST2^WT^ and ST2*^fl/fl^* CD3^+^ T cells labeled with CTV into the same allo recipient (Figure 8D). At d3 after alloHCT, donor ST2^WT^ CD4^+^ T cells in the spleen of allo recipients had increased Nur77 compared with the ST2*^fl/fl^* subset (Figure 8, E–G). IL-33–mediated increases in Nur77 were also observed when *Nur77-GFP* B6 donor CD4^+^ T cells were transferred into *Bm12 il33^+/+^* versus *Bm12 il33^–/–^* recipients (Supplemental Figure 5). By using the B6 CD4^+^ T cell to Bm12 recipients alloHCT model, we could assess how the absence of IL-33 modulated Nur77 upregulation in donor CD4^+^ T cells in response to a single MHCII mismatch (55, 56). Analysis of splenic donor T cells in *Bm12 il33^+/+^* recipients at d3 after alloHCT found increased Nur77 in the expanded CD4^+^Nur77^+^ subset compared with the same group of donor T cells isolated from *Bm12 il33^–/–^* recipients (Supplemental Figure 5, A–D). This population in *Bm12 il33^–/–^* recipients also had increased retention of CD62L (Supplemental Figure 5E). While there were no differences in donor CD4^+^ T cell numbers at d3 (Supplemental Figure 5F), decreases in donor CD4^+^ T cells were observed in the *Bm12 il33^–/–^* recipients at d7 (Supplemental Figure 5G). This decrease could be partially corrected by the delivery of rIL-33 (Supplemental Figure 5F). Mortality and morbidity were also reduced in this CD4-dependent GVHD model by the absence of IL-33 (Supplemental Figure 5, H and I).

**Figure 9:**
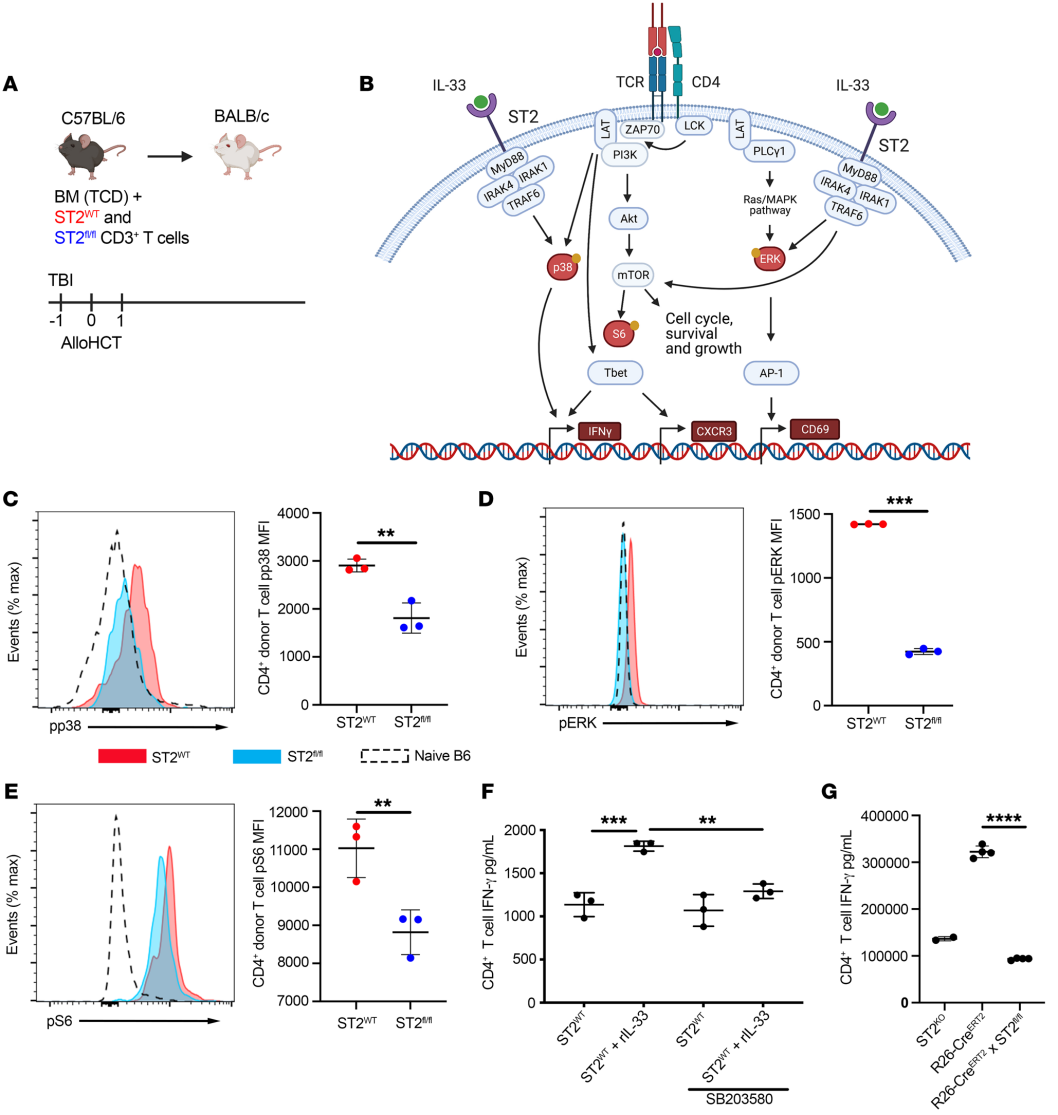
Alloantigen-driven TCR signaling networks are enhanced by IL-33 stimulation. (**A**) Model schematic. (**B**) Diagram of overlapping TCR and ST2-signaling pathways. (**C**–**E**) CTV-labeled CD3^+^ T cells from CD45.2^+^ *CD4-Cre*×*R26-LSL-YFP*×*St2^fl/fl^* B6 (1 × 10^6^) and *St2^+/+^* CD45.1^+^ B6 (1 × 10^6^) mice were cotransferred with 1 × 10^7^ WT B6 TCD-BM into lethally irradiated BALB/c recipients. ST2^WT^ (red) and ST2*^fl/fl^* (blue) CD4^+^ T cells from the same spleen were assessed by flow cytometry on d1. (**C**) Representative histograms of p-p38 and quantification of p-p38 MFI; (**D**) pERK expression and quantification of pERK MFI; (**E**) pS6 expression and quantification of pS6 MFI on d1 from ST2^WT^ (red) and ST2*^fl/fl^* (blue) donor CD4^+^ T cells (**C**–**E**). (**F**) Sorted naive CD4^+^ T cells from *St2^+/+^* B6 were stimulated in vitro with anti-CD3/CD28 beads, IL-12, IL-2, and anti–IL-4 for 4d, followed by a 3-hour rest and 24-hour IL-33 stimulation (or no stimulation) with or without p38 inhibition (SB203580). IFN-γ in supernatants was assessed by ELISA. (**G**) Sorted naive CD4^+^ T cells from *St2^+/+^* B6 were stimulated in vitro with anti-CD3/CD28 beads, IL-12, IL-2, anti–IL-4, IL-33, and TAM for 4 days, and IFN-γ in supernatants was determined by ELISA. Data in **C**–**F** are represented as mean ± SD with *n* = 3–4/group. Data are representative of 2 experiments. Data in **G** show *n* = 2–4/group. *P < 0.05; **P < 0.01; ***P < 0.001; ****P < 0.0001, Student’s *t* test (**C**–**E**); 1-way ANOVA (**F** and **G**).

We then investigated whether IL-33 modulated early TCR signaling by assessing phosphorylation of pathway intermediates involved in TCR signaling after alloHCT (Figure 9, A and B). Phosphorylation of kinases downstream of TCR and ST2 were measured in donor CD4^+^ T cells from ST2*^fl/fl^* and ST2^WT^ mice, which were labeled with CTV and cotransferred into the same lethally irradiated allo recipient (Figure 9, A and B, and Supplemental Figure 6, A and B). Splenocytes were assessed on d1 after alloHCT (Supplemental Figure 6, A and B). ST2^WT^ CD4^+^ T cells had increased levels of phosphorylated p38 (p-p38) and ERK as well as the MTORC1 target S6 compared with ST2*^fl/fl^* T cells from the same recipient (Figure 9, C–E). These findings establish that ST2 augments activation of pathways that are common with TCR signaling. We also show the importance of the p38 pathways to Th1 effector function, as p38 inhibition negates IL-33–mediated IFN-γ production by CD4^+^ T cells (Figure 9F). To demonstrate ST2-deficient T cells are not intrinsically defective from a lack of IL-33 stimulation early during T cell development, we used ST2 inducible knockout mice to delete ST2 at the time of activation and Th1 differentiation. Specifically, when B6 *R26-cre^ERT2^xSt2^fl/fl^* and *R26-cre^ERT2^* were Th1 skewed in vitro and treated with 4-hydroxytamoxifen (Tam) and rIL-33, *R26-cre^ERT2^* mice produced IFN-γ in response to IL-33, but Tam-treated *R26-cre^ERT2^xSt2^fl/fl^* mice, whose ST2 would be deleted, produced levels of IFN-γ comparable to those of *St2^–/–^* controls (Figure 9G). Thus, the observed differences are not due to a lack of ST2 signaling during development, but to the loss of IL-33 stimulation of donor T cells following alloHCT. In total, our data establish that early after alloHCT, IL-33 acts as a potent costimulatory signal that augments TCR signaling to support alloreactive CD4^+^ T cell activation, proliferation, and Th1 differentiation.

## Discussion

In the current studies, we provide mechanistic insights into IL-33–mediated CD4^+^ T cell immunobiology after alloHCT and build on our past observation that IL-33 is a clinically relevant, recipient-derived DAMP that drives lethal GVHD (29). Specifically, we reveal that FRCs in the SLOs are an important IL-33 source during the time of donor T cell expansion and the initiation of GVHD. IL-33 contributes to the generation of alloreactive Th1 cells by functioning as a costimulatory signal that acts directly on donor CD4^+^ T cells to augment TCR-related signaling pathways. Another important finding was that IL-33 promoted Th1 differentiation independently of IL-12. IL-33 has been described as a DAMP released from epithelial barrier tissues and perivascular regions of the lung to aid parasite clearance and contribute to allergy through the antigen-independent functions of type 2 innate lymphoid cells and Th2 cells (20, 57). In contrast, our findings suggest an important function for IL-33 in contributing to antigen-dependent activation and differentiation of alloreactive CD4^+^ T cells into Th1 cells. Our identification of IL-33 as a stromal cell–derived DAMP that is a costimulatory signal driving the direct activation and differentiation of alloreactive Th1 cells independently of the indirect PAMP-stimulated innate cytokine, IL-12, provides insights into targetable signals supporting GVHD initiation.

ST2 is a member of the IL-1R/TLR superfamily expressed constitutively or induced on a wide range of immune and nonhematopoietic cells (20, 58). Evidence is emerging supporting costimulatory roles for IL-1R/TLR ligands that directly promote T cell proliferation, survival, and differentiation (59–61). IL-1 family members, such as IL-33 and IL-1, can work synergistically with STAT stimulators to enhance T cell effector responses (62, 63). Likewise, IL-1β has been described as an adjuvant that boosts T cell expansion and survival following cognate antigen stimulation independently of IL-6 and CD28 signaling (17, 64). Recently, Matsuoka et al. demonstrated that MyD88 signaling in T cells was needed for donor T cell survival and Th1, Tc1, and Th17 differentiation leading to GVHD lethality (19). A lack of MyD88 in donor T cells also facilitated Foxp3^+^ Treg expansion after alloHCT to reduce GVHD lethality (19). ST2, like other IL-1R/TLR superfamily members, relies on MyD88 to convey IL-33 stimulation (20, 58, 65, 66). Our current findings regarding IL-33 promoting Th1 differentiation while limiting *Foxp3* and *Il10* expression are compatible with those of Matsuoka et al. (19). Likewise, the demonstration by these investigators that GVHD-promoting effects were independent of TLR2 and TLR7 support our conclusion that IL-33 is a critical T cell–stimulating DAMP after alloHCT. Griesenauer et al. have also demonstrated similar findings with *Myd88^–/–^* donor T cells conferring protection. While not pinpointing the specific ligand activating MyD88 signaling in T cells, they determined that the reduced GVHD was independent of IL-1R and TLR4 signaling, since knocking out these receptors on donor T cells did not provide the same protection (18). Our studies now provide a definitive ligand that can explain the phenotype of *Myd88*^–/–^ T cells in GVHD. When previous studies and our current studies are looked at in total, there is a strong indication that IL-33 released from recipient tissues ligates MyD88-dependent ST2 to act as a potent donor T cell costimulatory factor that supports Th1 differentiation and expansion while regulating the induction and function of Tregs.

Our data are divergent from findings using a lymphocytic choriomeningitis virus (LCMV) infection model in which *Stat4^–/–^* and *Tbet^–/–^* T cells did not upregulate ST2 following an LCMV challenge (67). Likewise, we had previously observed that IL-12 increased CD8^+^ T cell ST2 expression in vitro in a Tbet-dependent manner (28, 29). In the current studies, neutralization of IL-12p40 did not modulate ST2 expression on donor CD4^+^ T cells or reduce GVHD mortality over that observed when donor T cells lacked ST2. Given the rapid response of donor CD4^+^ T cells to IL-33, it is most likely that a low level of ST2 exists on naive T cells that initiate an immediate response to IL-33, with alloHCT-associated stimuli, such as IFN-γ, or TCR stimuli augmenting subsequent ST2 expression as these T cells are activated and proliferate. Identifying how ST2 is regulated on donor CD4^+^ T cells will be an important area of investigation moving forward that may lead to therapeutics that are able to limit the potency of the IL-33/ST2 axis after alloHCT.

Our data emphasize the importance of IL-33 for early activation and differentiation of CD4^+^ T cells into Th1 cells expressing Tbet and CXCR3 in the SLO. While we do not precisely rule out a role for IL-33 in other locations, such as the barrier tissues or GVHD target organs, our data establish a profound augmentation of IL-33 in the SLO after radiation exposure at times when donor T cells will predominantly be found in those SLO (68–70). It has been shown that radiation and alloHCT change the SLO structure and increase the relative density of FRC-like stromal cells that support GVHD development (31). Our data suggest these changes contribute to acute GVHD by augmented IL-33 availability in PDPN^+^CD31^–^ FRCs that then increase donor Th1 responses. This concept and our current findings align with past studies identifying the radiation-resistant nature of FRCs (71) and FRC of the splenic T cell zones being a critical source of IL-33 in LCMV infections (72). In T cell responses to LCMV, however, the IL-33–dependent phase of virus-reactive CD8^+^ T cell expansion begins after d4 after infection following priming and a first phase of expansion (72, 73). In contrast, IL-33 influence on donor CD4^+^ T cells starts at d1 and continues over several days. This difference may be due to recipient conditioning rapidly augmenting FRC IL-33 and triggering necrotic cell death as well as generating signals that promote IL-33 release or ST2 expression on alloreactive CD4^+^ T cells. Other factors potentially accounting for differing early IL-33 functions are the high number of alloreactive CD4^+^ T cells present at d1 of alloHCT or unique expression kinetics for IL-33. IL-33 is depleted in the SLO by d3 after LCMV infection, yet FRC frequency is not altered, suggesting that prestored nuclear IL-33 is released during viral infections (73). Our current data indicate that the conditioning necessary for alloHCT instead causes an augmented early and sustained presence of IL-33 in the SLO that initiates and promotes pathological Th1 alloimmune responses leading to GVHD.

Our studies raise several outstanding issues regarding IL-33 expression and release. First, how is IL-33, sequestered in the nucleus, being released from FRCs to stimulate Th1 responses, and can this process be modulated to control GVHD? Second, our data using ST2-deficient donors and recipients lacking IL-33 clearly establish that IL-33 is important after alloHCT to promote Tbet induction and CXCR3 expression that initiates Th1 cell infiltration into GVHD target tissues. Yet IL-33 is upregulated by alloHCT in epithelial cells and mesenchymal cells of barrier tissues, particularly the SI (29). Compelling work by Koyama et al. has demonstrated that nonhematopoietic recipient APCs in the target tissues are sufficient for promoting alloreactive donor T cell expansion to produce GVHD (25, 74). Future studies utilizing inducible ST2 deletion on donor T cells or IL-33 deletion in the SLO or GVHD target tissues will be important for showing what the roles for IL-33 are at these sites over the course of GVHD.

Numerous factors influence the lineage-specific differentiation of naive CD4^+^ T cells; these include the type of APC, concentration of antigen, duration of TCR ligation, costimulatory signals, and local cytokine environment (75). Early amplification of Tbet transcription involves p38 (76, 77), a signaling pathway activated by IL-33 ligation of ST2 (78). Several studies have suggested that Th1 cells preferentially differentiate in response to high-affinity antigen or high antigen dose, whereas lower affinity antigen and low antigen dose favor Th2 responses (79, 80). Weak affinity peptides used to stimulate TCR-transgenic T cells or low concentration of their cognate peptide induce Th2 differentiation through transient ERK activation, which is associated with IL-4 production and Gata3 expression (81–83). In contrast, high concentrations of cognate antigen or high-affinity peptide stimulation of TCR-transgenic T cells causes strong and prolonged ERK activation to suppress Gata3 expression (83). We show that IL-33 augments Tbet^+^ T cells during a CD4^+^ T cell response to alloantigen; we also show that IL-33 stimulation intensifies p38, ERK, and s6 phosphorylation in vivo. Together, these data suggest that IL-33 boosts TCR signaling to support CD4^+^ differentiation into Th1 cells. While alloantigen is ubiquitous in GVHD, the antigen affinity of alloreactive T cells causing GVHD is poorly defined. Our data using the *TEa* TCR suggest IL-33 can augment CD4^+^ T cell responses at low antigen doses, but it is difficult to determine whether this mechanism serves to boost weak TCR signals to diversify and broaden the alloresponse or is acting to complement strong TCR signals to support the survival of potent alloreactive clones after alloHCT (84).

GVHD occurs even if recipient cells lack the CD28 ligands CD80 and CD86 and is ablated only if donor APCs can present antigen to donor T cells (17, 74, 85). This suggests that there are recipient signals, such as IL-33, that provide the costimulation necessary for donor T cell activation. We identified IL-33 as upregulating the early activation marker CD69 in allo, but not syn, recipients. This suggested that CD69 was being upregulated in response to TCR and IL-33 stimulation. Yet CD69 can be induced by inflammatory stimuli, such as IFN-γ or innate cytokines released following TLR ligation (52, 54). However, we also found increased expression of Nur77 in ST2-competent CD4^+^ T cells compared with ST2-deficient ones in IL-33^+^ allo hosts. This was paralleled by increased Nur77 in CD4^+^ T cells in allo IL-33^+^ Bm12 recipients relative to those deficient for IL-33. The expression of Nur77 is dependent on TCR engagement and simultaneous costimulation, since anti-CD3 stimulation alone does not upregulate Nur77 (86). Thus, our data support the conclusion that recipient IL-33 acts as a costimulatory signal that augments TCR signaling pathways to support early activation and Th1 differentiation.

Despite evolving immunoprophylaxis regimens after alloHCT, donor T cell responses to MHC mismatches or miH antigens on matched MHC results in GVHD in 30% to 70% of recipients (4). It is well appreciated that the excessive expansion of alloreactive Th1 cells producing high levels of the proinflammatory cytokines IFN-γ and TNF-α is central to GVHD pathology. Interestingly, IL-33 promoted early Th1 differentiation and GVHD lethality independently of IL-12. This finding suggests that the combined targeting of IL-33 and IL-12 using antibodies undergoing clinical testing (87, 88) immediately after alloHCT would target 2 distinct Th1-driving pathways and thus be highly effective in limiting early donor Th1 responses. Our data indicate that IL-33 is not strongly involved in LIP, which is supported by IL-7 (89). IL-7 induces expression of the gut-homing integrin α_4_β_7_ and increases naive T cell homing to the SI (89). This may account for the observed IL-33–independent infiltration of donor T cells in syn recipients. We have shown that B6 *St2^–/–^* T cells retain the ability to clear BALB/c A20 B cell lymphomas, even though they are poor inducers of GVHD (29). Thus, targeting of IL-33 or ST2 signaling is not expected to disrupt T cell reconstitution or graft-versus-leukemia (GVL) responses. It is also of considerable interest that St2*^fl/fl^* donor CD4^+^ T cells have a gene transcript suggesting a regulatory phenotype and that *il33^–/–^* recipients have increased IL-10 in the serum at d7. These findings suggest that in the absence of IL-33 augmentation of TCR signaling, alloreactive donor T cells are prone to skewing toward more regulatory subsets, potentially Tregs or Tr1 cells. Targeting IL-33 or ST2 signaling to increase regulatory cells could have considerable therapeutic implications for patients receiving alloHCT, as it would be expected to lower GVHD risk. Augmenting regulatory responses during alloHCT would also aid tolerance induction for treating autoimmune disease or limiting the need for immunosuppression after solid organ transplantation.

## Methods

### Mice.

C57BL/6 (B6; H2-K^b^), Bm12, BALB/c (H2-K^d^), B6 CD45.1^+^, B6 CD90.1^+^, B6 Nur77-GFP, B6 *TEa* Tg, and B6 *R26-cre^ERT2^* mice were purchased from The Jackson Laboratory. *Il33^–/–^* mice were from Susumu Nakae (University of Tokyo, Tokyo, Japan; ref. 90). *CD4-cre*×*R26-LSL-YFP* mice (C57BL/6; *CD4^Cre^*) were from Dario Vignali (University of Pittsburgh). Bm12 *il33^–/–^* mice were generated by backcrossing Bm12 mice 6 times onto a B6 *il33^–/–^* background. *St2^–/–^* mice were generated in BALB/c (91) and backcrossed 10 times onto the B6 background. B6 *St2^fl/fl^* mice were provided by Giorgio Trinchieri (National Cancer Institute, Bethesda, Maryland, USA), crossed to *CD4^Cre^* to generate *CD4^Cre^*x*St2^fl/fl^* mice, and crossed to B6 *R26-cre^ERT2^* to generate B6 *R26-cre^ERT2^*x*St2^fl/fl^*. All mice used were 6 to 10 weeks old at the time of initiation of experimental procedures. All mice were bred and/or maintained in specific pathogen–free animal facilities.

### AlloHCT and GVHD.

Female recipient mice were exposed to lethal TBI (B6: 1100 cGy; BALB/c: 800 cGy; BM12: 900 cGy over 2 doses separated by 3 hours) on d–1 prior to alloHCT. On d0, B6 and BALB/c recipient mice were given 1 × 10^7^ TCD allo BM cells alone or with 2 × 10^6^ CD3-purified splenic allo T cells; Bm12 recipient mice were given 1 × 10^7^ TCD allo BM cells alone or with 1 × 10^5^ CD4-purified splenic allo T cells i.v. Survival and weights were recorded, and clinical score assessment was performed as described (29, 78).

### Animal treatments.

BALB/c mice were treated with PBS or 0.5 μg recombinant mouse IL-33 (BioLegend) i.p. every day for 5d. The first dose was on d3 after alloHCT through d7. For α–IL-12p40 treatments, mice were treated i.p. with 500 μg/mouse/d or IgG as control starting on d–1, the same day as the TBI, and every 3 days after (d2, d5, and d8 for survival studies and d2 and d5 for mechanism studies). For Eα_52–68_ peptide treatments, mice were given an i.p. injection with either 0.1 μg, 1 μg, 10 μg, or 100 μg in PBS on d0. The weight and survival of treated animals were monitored. In survival studies, survival was defined as death or euthanasia after sustained weight loss greater than 30% to 35% of the starting weight.

### Isolation of FRCs, lamina propria lymphocytes, splenocytes, and LN cells.

Lamina propria lymphocytes (LPLs) were isolated from the SI using the MACS Mouse Lamina Propria Dissociation Kit (Miltenyi Biotec) according to the manufacturer’s instructions as described (78). Following LPL isolation, T cells were enriched using a debris removal kit (Miltenyi Biotec) and density gradient centrifugation at 1000*g* before analysis. FRCs were isolated from LN and spleen digests, with spleen preparations enriched for nonhematopoietic stromal cells using negative selection with CD45 microbeads (Miltenyi Biotec). Single-cell homogenates of splenocytes and lymph nodes were generated for flow cytometric assessment by mechanical disruption. Detailed methods for isolation of FRCs are provided in the Supplemental Methods.

### Ex vivo FRC culture and MLR.

FRCs were isolated from the LNs (axillary, brachial, and inguinal) from B6 mice with negative enriched for nonhematopoietic stromal cells using CD45 microbeads and FRCs using CD31 microbeads (Miltenyi Biotec). Purified FRCs were then radiated (3.5 Gy) or left untreated and plated at 4000/well in a 24-well plate. After settling for 24 hours, they were cultured with T cell–depleted, irradiated (3.5 Gy) B6 splenocytes at 2.5 × 10^5^/well and BALB/c *St2^+/+^* or *St2^–/–^* T cells 1 × 10^6^/well for 3 days. Total donor CD4^+^ T cells were determined by flow cytometry.

### Flow cytometry analysis and phosphoflow.

Detailed methods and resource information, including clone numbers and antibody sources, are provided in the Supplemental Methods.

### RNA-Seq and bioinformatics analyses.

B6 CD45.1^+^ (ST2^WT^) and B6 CD45.2^+^ *CD4-Cre*×*R26-LSL-YFP*×*St2^fl/fl^* (ST2*^fl/fl^*) donor splenic CD4^+^ T cells were enriched from CD90.1^+^ B6 or H2-K^d^ BALB/c recipient mice (*n* = 4/group) using negative depletion with Dynabeads (Life Technologies). The cells were stained for CD3, CD4, CD90.1, H2-K^d^ CD45.1, and CD45.2 and sorted for ST2^WT^ and ST2*^fl/fl^* CD4^+^ populations on a FACSAria II, directly into SmartSeq Low-Input RNA Kit lysis buffer for library prep and RNA-Seq. Detailed analysis procedures are provided in the Supplemental Methods. Data were deposited in the NCBI’s Gene Expression Omnibus database (GEO GSE197775).

### Immunofluorescent histology.

Optimal cutting temperature compound–embedded (Thermo Fisher Scientific) frozen spleens were sectioned, placed on glass slides, and stained as described (29). Further details are provided in the Supplemental Methods.

### Statistics.

Statistical details of each experiment can be found in the figure legends. Data are presented as mean ± SD, and *n* refers to biological replicates. *P* < 0.05 was considered statistically significant. GraphPad PRISM 9.0c software was used for statistical analyses. Littermate controls were used throughout the study. The Shapiro-Wilk test was employed throughout the study, and samples assessed did not show significant evidence for deviation from normality. Data significance was analyzed using a 2-tailed, unpaired or paired Student’s *t* test in cases where 2 groups were being compared. In cases where more than 2 groups were being compared, 1-way or 2-way ANOVA was used with Šidák’s test to correct for multiple comparisons.

### Study approval.

All procedures completed at the University of Pittsburgh were approved by the Institutional Animal Care and Use Committee of the University of Pittsburgh (protocol 21079668) and complied with the NIH’s *Guide for the Care and Use of Laboratory Animals* (National Academies Press, 2011). All experiments by the Luther lab were authorized by the Swiss Federal Veterinary Office (VD3196x1).

## Author contributions

GKD, BRB, JPG, ACP, WS, JAV, SAL, and HRT conceptualized and designed the research. GKD, LRM, AL, AGDP, ACP, JAV, and HRT performed the experiments. GKD, LRM, AL, AGDP, ACP, WS, JAV, SAL, and HRT analyzed the data and interpreted the results. GKD, LRM, AL, and HRT prepared the figures. GKD, BRB, JPG, WS, JAV, SAL, and HRT drafted and edited the manuscript.

## Supplementary Material

Supplemental data

## Version 1 05/03/2022

In-Press Preview

## Version 2 06/15/2022

Electronic publication
