# Supplementary material for: IL-33 acts as a costimulatory signal to generate alloreactive Th1 cells in graft-versus-host disease
Source: J Clin Invest. 2022 Jun 15;132(12):e150927. doi: 10.1172/JCI150927 (PMC9197517; doi:10.1172/JCI150927)
Supplement: Supplemental data [file jci-132-150927-s163.pdf]

Supplemental Methods, Data Figures, and Table for Dwyer et al. "IL-33 acts as a novel costimulatory agent to generate alloreactive Type 1 T helper cells"

Supplemental Material Summary

Supplementary Methods provide a detailed description of flow cytometry, phosphoflow, immunofluorescent histology, general cell isolation procedures, RNA-Seq and bioinformatics analyses, and T cell protein isolation and analysis methods. References in the Supplementary Methods are provided. **Fig. S1** shows the gating strategy for identifying $ST2^{WT}$ and $ST2^{KO}$ donor $CD4^+$ T cells and co-transfer $ST2^{WT}$ and $ST2^{KO}$ donor $CD4^+$ T cell counts, CD44 and Tbet MFI. **Fig. S2** shows the ST2 expression of syngeneic donor $CD4^+$ T cells on d3, 5, and, 7 of lymphopenic-induced expansion. **Fig. S3** shows the characterization of the *CD4-Cre x R26-LSL-YFP x* $St2^{fl/fl}$ ($ST2^{fl/fl}$) mouse and CD69 protein expression in IL-33 stimulated $CD4^+$ T cells *in vitro.* **Fig. S4** shows the GSEA enriched in the $ST2^{WT}$ $CD4^+$ T cells for mitochondrial activation and Myc targets and the serum levels of IFNγ and IL-10 in $il33^{-/-}$ recipients compared to the $il33^{+/+}$ recipients day 7 post-alloHCT. **Fig. S5** demonstrates the gating strategy for identifying $Nur77^+$ donor $CD4^+$ T cells and shows augmentation of early response gene, Nur77, in the Bm12 GVHD model on d3 as well as improved survival and clinical scores of Bm12 $il33^{-/-}$ compared to Bm12 WT mice. **Fig. S6** shows the gating strategy to identify $ST2^{WT}$ and $ST2^{fl/fl}$ donor $CD4^+$ T cells on day 1 post-alloHCT for phosphoflow analysis. **Table S1** lists gene signatures used in RNAseq analysis.

Flow cytometry analysis

Isolated cells were stained using the following antibodies to indicated molecules (Clone #): BD Bioscience: CD45.1 (A20), CD4 (RM4-5), CD44 (IM7), Tbet (O4-46), Gata3 (L50-823), Rorγt (Q31-378), Foxp3 (MF23), ST2 (U29-93), CD90.1 (OX-7), $H2\text{-}K^d$ (SF1-1.1.1) and Vβ6 (RR4-7); BioLegend: CD25 (PC61), Ki67 (16A8), GFP (FM264G), CXCR3 (CXCR3-173), CD62L (MEL-14), CD69 (H1.2F3), CD8 (53-6.7), CD3ε (17A2), and CD45.2 (104). For stromal cell analysis: Biolegend: CD31 (390), TER-119 (TER-119), Podoplanin (8.1.1), CD45 (30-F11); Generated In house: CD157 (BP3.4); R&D: IL-33 (AF3626). Data were acquired using an LSR Fortessa (BD Biosciences) or Aurora (Cytek Biosciences) and analyzed using FlowJo (Tree Star, Ashland, OR). Intracellular staining was carried out using Foxp3/Transcription Factor staining buffer set (eBioscience; San Diego, CA). Live/dead exclusion was completed using E506 Viability Dye (Invitrogen) or Zombie Aqua fixable viability kit (Biolegend).

Phosphoflow

Pan T cells from CD45.2$^+$ *CD4-CrexR26-LSL-YFPxSt2$^{fl/fl}$* B6 ($1x10^6$) and *St2$^{+/+}$* CD45.1$^+$ B6 ($1x10^6$) mice were labeled with CTV and adoptively transferred with $1x10^7$ WT B6 TCD-BM into lethally irradiated BALB/c recipients. On d1 single-cell splenocytes suspensions were prepared. For surface and intracellular staining, cells were fixed and permeabilized in saponin-based Perm/Wash buffer (Cat#: 554732, BD Biosciences) supplemented with 1.5% paraformaldehyde (PFA) and blocked with Fc receptor antibody (CD16/32, 93; BioLegend) prior to staining with surface antibodies (listed above) and fluorochrome-conjugated phospho-antibodies (BioLegend): phospho-p38 MAPK (Thr180/Tyr182; A16016A), phospho-S6 (S235/236, A17020B), or phospho-Erk1/2 (T202/Y204, 4B11B69).

Immunofluorescent histology

Optimal cutting temperature compound–embedded (Thermo Fisher Scientific) frozen spleens were sectioned (6 μm), placed on glass slides, and stained for H2-K^d (eBioscience, 34-1-2S), Desmin (Invitrogen, PA5-16705), CD31 (In house generated, GC-51), and IL-33 (R&D Systems, AF3626), and then fluorochrome-conjugated and species-specific secondary antibodies as described (1). Primary antibodies were followed by secondary antibodies conjugated to Alexa Fluor 555 (Donkey anti-Goat IgG, Invitrogen, A21432), Alexa Fluor 488 (Donkey anti-Rat IgG, Invitrogen, A-21208), Alexa Fluor 647 (Donkey anti-rabbit IgG, Invitrogen, A31573), Cyanine Cy3 (Donkey anti-goat IgG, Jackson ImmunoResearch Labs, Inc., 705-165-147), and Alexa Fluor 647-conjugated Streptavidin (Jackson ImmunoResearch Labs, Inc., 016-60-084). Nuclei were stained with 4′,6-diamidino-2-phenylindole (DAPI; Sigma). Sections were visualized following whole slide image capture using a Zeiss Axio Scan.Z1 scanner, on the 40x objective, utilizing a 16-bit color sCMOS camera (Hamamatsu Photonics), and HXP-120V metal halide excitation source or monochrome camera, and appropriate filters, or Hamamatsu NanoZoomer S60 on the 40x objective, utilizing a 8-bit color CMOS camera (Hamamatsu Photonics). Images were acquired and processed using ZEN Blue 3.1 software or NDP-view2 software. Image analysis was done with Image J (Fiji). All czi file from ZEN Blue 3.1 were exported from ZEN Blue 3.1 as tiffs.

Isolation of fibroblast reticular cells (FRCs)

Splenic stromal cells were isolated based on the protocol from Alexandre et al. (2). Briefly, spleens were recovered and perfused with 1 ml RPMI 1640 containing 2% FBS, Collagenase D (2 mg/ml) DNase I (0.1 mg/ml) and Dispase II (0.8 mg/ml). Then spleens were incubated for 35 minutes (min) at 37°C in a water bath with a change of digestion media after 25 min. Spleens were mixed

thoroughly to obtain a single-cell suspension. Red blood cells were lysed and recovered cells washed with FACS buffer (1x phosphate-buffered saline, 2% bovine serum albumin, and 5 mM EDTA). To enrich splenic stromal cells, recovered cells were depleted of $CD45^+$ cells using CD45 microbeads and LS columns (Miltenyi Biotec), and then washed and stained for FACS analysis. Lymph node stromal cells were recovered from axillary, brachial, and inguinal LN. Lymph nodes were cut into small pieces and incubated in RPMI 1640 containing Collagenase P (0.2 mg/ml), DNase I (0.01mg/ml) and Dispase II (0.8 mg/ml) for 30 min at 37°C in water bath with a change of digestion media after 20 min. Cell suspensions were mixed thoroughly, then washed in FACS buffer and stained for FACS analysis.

RNA-Seq and bioinformatics analyses.

Libraries were prepared from isolated RNA using Nextera XT DNA library prep kits, and RNA sequencing was performed on Illumina NextSeq500 by the Health Sciences Sequencing Core at the University of Pittsburgh. Raw sequencing data were processed using CLC Genomics Workbench 20.0.3 (QIAGEN Inc., https://digitalinsights.qiagen.com) for quality control and aligned to the *Mus musculus* genome version GRCm38.p6. Reads assigned to each gene underwent TMM normalization and differential expression analysis was performed using *edgeR* within CLC Genomics to compare $ST2^{WT}$ versus $ST2^{fl/fl}$ donor T cells. The top differentially expressed genes were filtered by adjusted p-value $q<0.05$ and fold-change greater than 1.5 for subsequent downstream pathway analysis. The relative expression shown in heatmaps was calculated as the fragments per kilobase exon per million mapped reads value for each sample divided by the mean expression of that gene in all samples per each experiment. GSEA from the Broad Institute (http://www.broad.mit.edu/gsea) was used to calculate the enrichment of genes in each set.

Protein isolation and analysis methods.

$CD4^+$ T cells were enriched from *St2*$^{+/+}$ B6 mice using negative depletion with a Naïve $CD4^+$ T cell isolation kit (Miltenyi) and were stimulated *in vitro* with anti-CD3/CD28 plate-bound antibodies with or without rIL-33 (5ng/ml) co-stimulation. T cells were lysed through heating in strong sodium deoxycholate detergent buffer as described (**3**). Following sonication to homogenize samples and shear the DNA, the crude protein extracts were enzymatically digested with Lys-C and trypsin, followed by peptide micro-purification on styrene divinylbenzene-reversed phase sulfonate StageTips to remove detergent and salts. Eluted peptides were analyzed using an UltiMate 3000 RSLC nanoflow chromatography system (Thermo Fisher Scientific) coupled to a

Q-Exactive HFX mass spectrometer (Thermo Scientific), by separation of the peptides using a 120min gradient of acetonitrile-containing mobile phase, on a 50 cm analytical C-18 column, in-house packed with 2µm porous silica beads. Online mass spectrometry analysis was performed in data-dependent acquisition mode. Raw MS files were processed with MaxQuant for protein identification through database search, and protein relative quantification by analysis of peptides MS signal intensity.

In vitro Th1 skewing and ELISA assays

CD4$^+$ T cells were enriched from *St2*$^{+/+}$ B6 mice using negative depletion with Dynabeads (Life Technologies). 3 hours prior to plating, plates were incubated with anti-CD3 and anti-CD28 (5µg/ml). T cells were Th1 skewed with rIL-12, rIL-2 and anti-IL-4 for 4 days, followed by a 3hr rest and 24hr rIL-33 (1ng/ml) stimulation (or no stim) with or without p38 inhibitions. The p38 MAPK inhibitor SB203580 (Tocris/Bio-Techne; Minneapolis, MN) was used at 5µM. At the end of culture, supernatants were snap frozen and stored at –80°C until use. Samples were batch thawed and diluted at no less than 1:10. Samples were run in triplicate and levels of IFNγ were measured by ELISA per manufacturer (R+D Systems) specification. Serum assessments for IL-10 and IFNγ were completed using ELISA per manufacture (R+D Systems) specifications.

Relevant References:

1. Reichenbach DK, Schwarze V, Matta BM, Tkachev V, Lieberknecht E, Liu Q, et al. The IL-33/ST2 axis augments effector T-cell responses during acute GVHD. *Blood.* 2015;125(20):3183-92.
2. Alexandre YO, Schienstock D, Lee HJ, Gandolfo LC, Williams CG, Devi S, et al. A diverse fibroblastic stromal cell landscape in the spleen directs tissue homeostasis and immunity. Sci Immunol. 2022;7(67):eabj0641.
3. Kulak NA, Pichler G, Paron I, Nagaraj N, and Mann M. Minimal, encapsulated proteomic-sample processing applied to copy-number estimation in eukaryotic cells. Nat Methods. 2014;11(3):319-24.

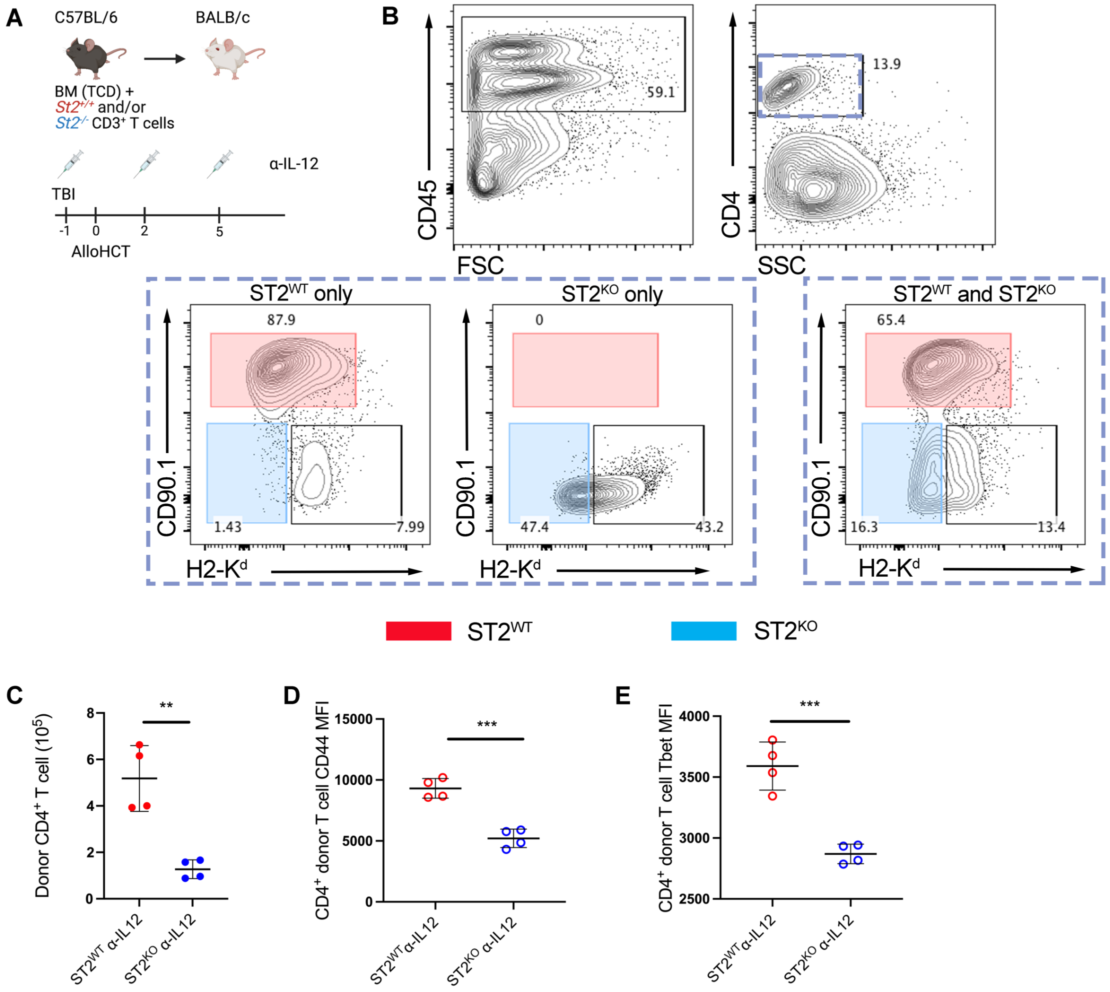

Supplemental Figure 1. *St2*$^{-/-}$ donor CD4^{+} T cells fail to expand at the same rates as *St2*$^{+/+}$ donor CD4^{+} T cells in response to alloantigen in the absence of IL-12 signaling. ***A-C,*** On d-1 BALB/c recipient mice received anti(α)-IL-12p40 or IgG as control and received lethal TBI. On d0, mice received 1x10^{7} B6 TCD-BM with a combination of CD90.1^{-}*St2*$^{-/-}$ (ST2^{KO}) (1x10^{6}) and CD90.1^{+}*St2*$^{+/+}$ (ST2^{WT}) (1x10^{6}) CD3^{+} T cells. Donor CD4^{+} T cells from the same spleen were compared at d7 by flow cytometry. ***A***, Schematic of B6 to BALB/c GVHD model as it relates to antibody (IgG or α-IL-12p40) treatments. ***B,*** Representative flow plot gating to identify CD4^{+}CD90.1^{+}H2-K^{d-} ST2^{WT} (red) and CD4^{+}CD90.1^{-}H2-K^{d-} ST2^{KO} (blue) donor CD4^{+} T cells in the same spleen (co-transfer) or separate spleens. ***C***, Co-transferred donor CD4^{+}CD90.1^{+} ST2^{WT} and CD4^{+}CD90.1^{-}H2-K^{d-} ST2^{KO} cell counts in recipients that received α-IL-12p40. ***D***, MFI of CD44 on co-transferred donor ST2^{WT} and ST2^{KO} CD4^{+} T cells from the same recipient spleen. ***E,*** MFI of Tbet on co-transferred donor ST2^{WT} and ST2^{KO} CD4^{+} T cells from the same recipient spleen. Data in ***B-E*** indicate mean+/-SD, *n*=4/group. *, P<0.05, **, P<0.01, ***, P<0.001, Student's *t* test (***C-E***).

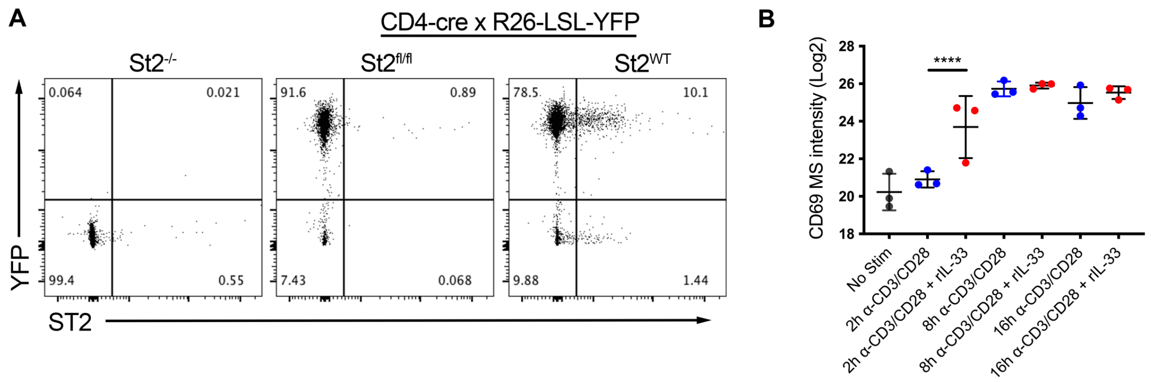

Supplemental Figure 2. CD4$^+$ T cells from *CD4-Cre x R26-LSL-YFP x St2$^{fl/fl}$* mice do not express ST2 and IL-33 induces early activation marker protein expression. *A,* B6 *St2$^{-/-}$, CD4-Cre x R26-LSL-YFP x St2$^{fl/fl}$*, and *CD4-Cre x R26-LSL-YFP* mice were treated with 0.5 ug IL-33 for 10 days to expand the CD4$^+$ST2$^+$ population. On day 11 splenocytes were harvested and assessed by flow cytometry. Plots are representative of two mice per group, gated on CD4$^+$CD25$^+$CD44$^+$ T cells. ***B,*** Mass spectrometry analysis of CD69 protein expression in naïve CD4$^+$ T cells following activation with anti-CD3/CD28 plate-bound antibodies with or without rIL-33 co-stimulation. Data in ***A*** *n*=2/group. Data in ***B*** indicate mean+/-SD, *n*=3/group. ****, P<0.0001, one-way ANOVA (***B***).

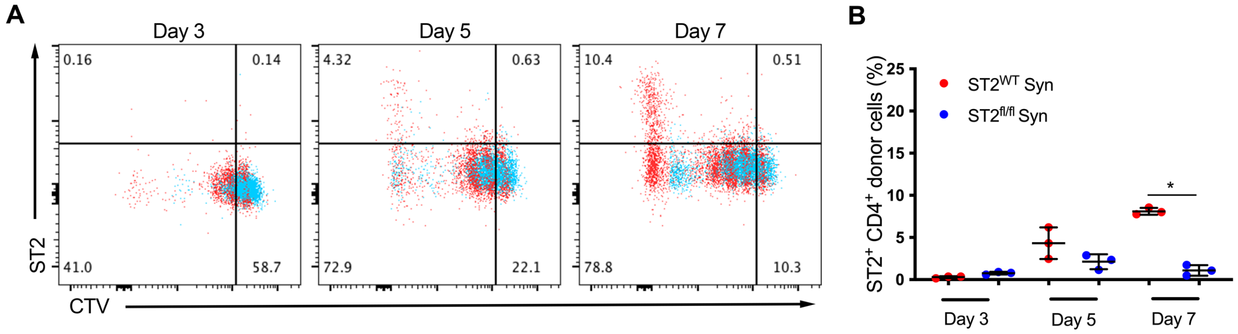

Supplemental Figure 3. Upregulation of ST2 by proliferating syngeneic CD4^{+} T cells. B6 (syngeneic; syn) recipient mice received lethal TBI (11Gy) on d-1. On d0 B6 recipient mice received 1x10^{7} WT B6 TCD-BM with 1x10^{6} CD3^{+} T cells from CD45.2^{+} *CD4-Cre x R26-LSL-YFP x St2$^{fl/fl}$* (ST2$^{fl/fl}$) and 1x10^{6} CD3^{+} T cells from *St2$^{+/+}$* CD45.1^{+} (ST2^{WT}) B6 mice. T cells were labeled with CTV prior to adoptive transfer. T cells were harvested from the spleen on d1, 2, 3, 5, and 7 post-adoptive transfer ***A,*** Representative flow plots of ST2 expression on donor CD4^{+}CD45.1^{+}H2-K^{d-} ST2^{WT} (red, quadrant frequencies) and CD4^{+}CD45.2^{+}H2-K^{d-}YFP^{+} ST2$^{fl/fl}$ (blue) cells from isolated from the spleen of the same syn recipient. ***B,*** Frequency of ST2 on donor CD45.1^{+} or CD45.2^{+} CTV^{lo} donor T cells on d3, 5, and 7. *, $P<0.05$, two-way ANOVA.

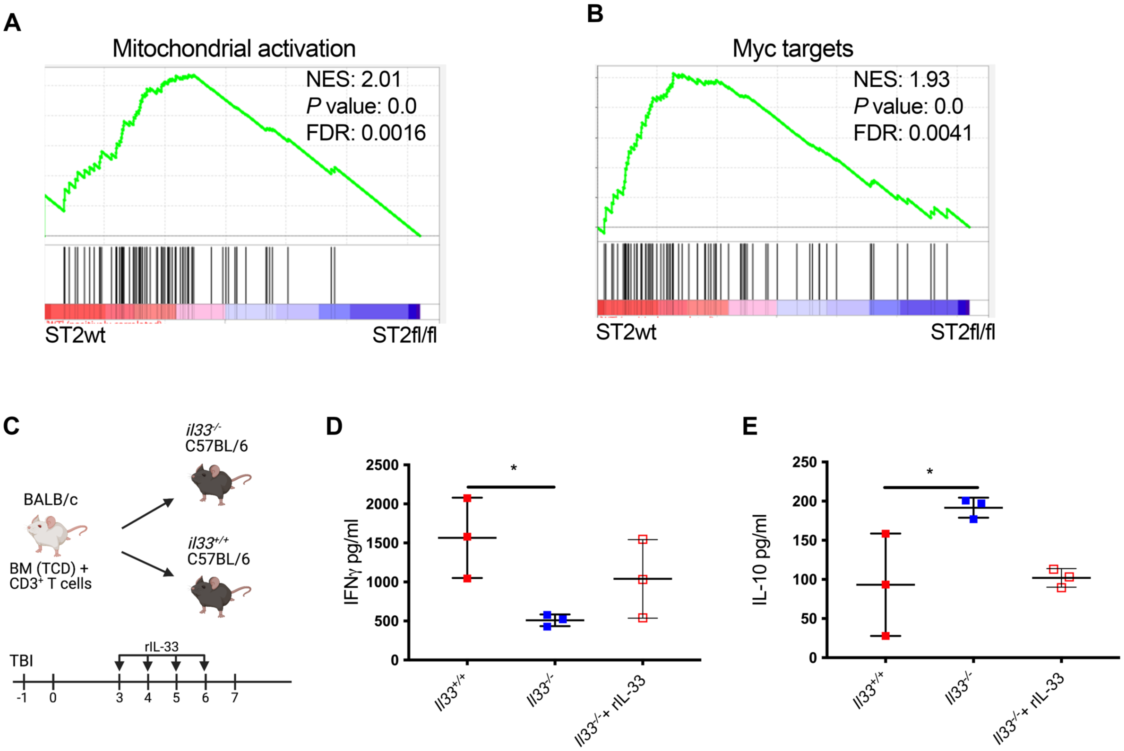

Supplemental Figure 4. Recipient IL-33 drives T cell metabolism and cell cycle gene expression and suppresses regulatory cytokine production. ***A,B,*** Leading edge plots of GSEA of ST2^{WT} (red) or ST2$^{fl/fl}$ (blue) donor CD4$^+$ T cells from an allogeneic recipient RNA-seq (described in Fig 6 E-I) compared with transcriptional profiles of Mitochondrial activation and Myc targets. ***C-E,*** On d-1 CD90.1$^+$ *il33*$^{-/-}$ or CD90.1$^+$ *il33*$^{+/+}$ B6 recipient mice received lethal TBI. On d0, mice received 1x10^7 CD90.2$^+$ BALB/c TCD-BM with 2x10^6 CD90.2$^+$ BALB/c CD3$^+$ T cells. Mice were treated with rIL-33 (from d +3 to +6 after HCT; 0.5 µg/mouse/d) or phosphate-buffered saline (PBS) as control. ***C***, Schematic of BALB/c to B6 GVHD model as it relates to rIL-33 treatments. ***D,*** Donor serum was collected on d7 and assessed for systemic IFNγ by Luminex array. ***E,*** Donor serum was assessed for systemic IL-10 by Luminex array. FDR, false discovery rate. NES, normalized enrichment score. Data in ***A,B,*** indicate mean+/-SD, *n*=4/group. Data in ***D,E,*** indicate mean+/-SD, *n*=3/group.*, P<0.05, one-way ANOVA (***D,E***).

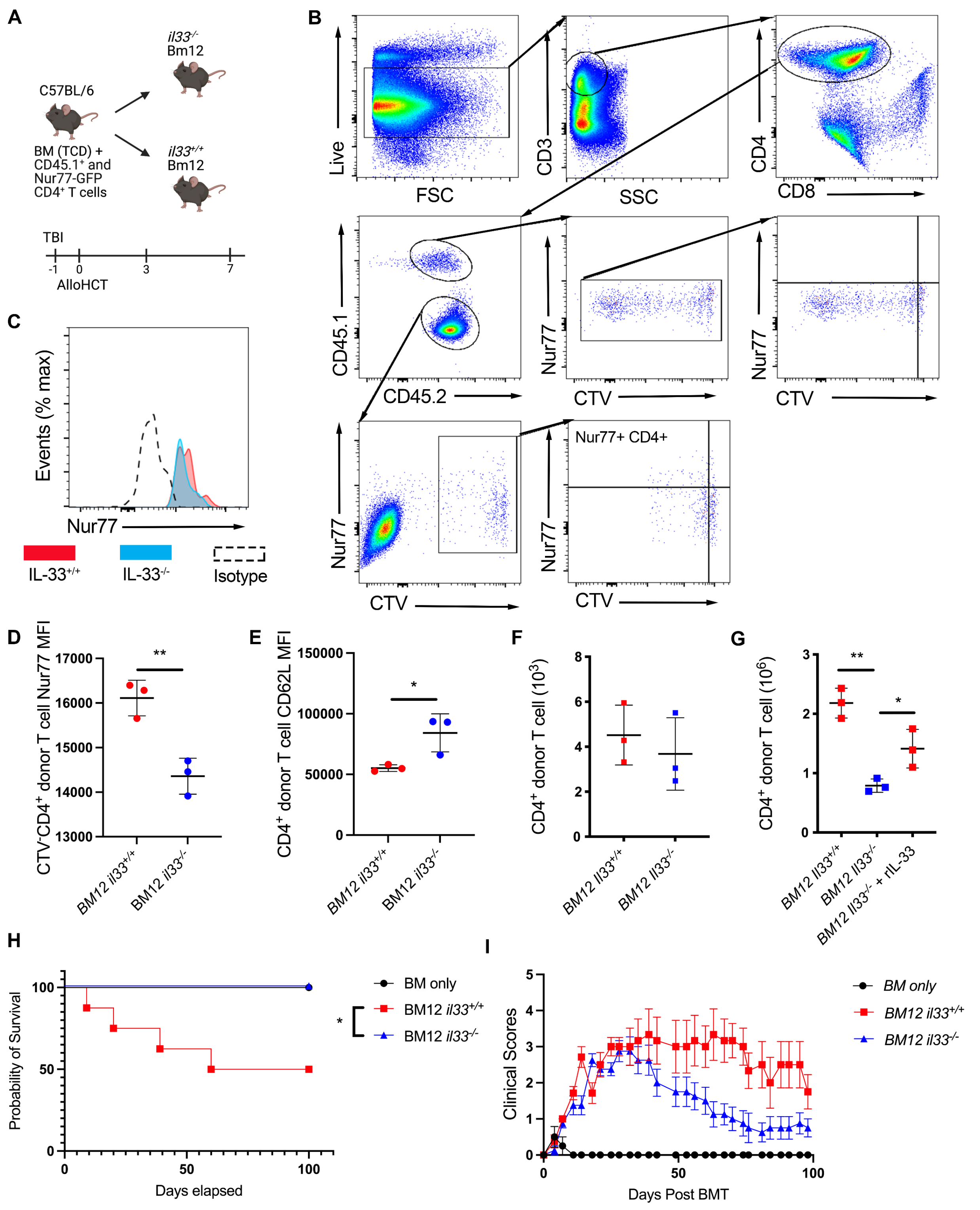

Supplemental Figure 5. IL-33 stimulation augments immediate-early response gene, Nur77, expression in CD4+ and supports GVHD lethality in a CD4+ T cell-driven GVHD model. Bm12

il33$^{+/+}$ and Bm12 *il33*$^{-/-}$ recipient mice received lethal TBI (9Gy) on d-1. On d0 recipient mice received 1x10^{7} WT Bm12 *il33*$^{+/+}$ TCD-BM with (***A-D***) 5x10^{4} *Nur77-GFP* B6 CD45.2^{+} CD4^{+} T cells and 5x10^{4} CD45.1^{+} B6 CD4^{+} T cells or (***E-I***) 1x10^{5} B6 CD4^{+} T cells. ***A-D***, Donor T cells were labeled with CTV prior to adoptive transfer. T cells were harvested from the spleen on d3 and d7 post-alloHCT. ***A,*** Schematic of mechanistic examination of the impact of recipient IL-33 stimulation of donor CD4^{+} T cells during GVHD initiation. ***B,*** Representative flow plot gating to identify donor CD45.1^{+}CD4^{+}Nur77-GFP^{-} and CD45.2^{+}CD4^{+} Nur77-GFP^{+} T cells. ***C,*** Representative histogram of donor CD4^{+} T cells Nur77-GFP expression from Bm12 *il-33*$^{+/+}$ and Bm12 *il-33*$^{-/-}$ recipient spleens d3. ***D,*** Quantification of Nur77-GFP MFI on d3. ***E,*** Quantification of CD62L MFI on Nur77-GFP^{+} donor CD4^{+} T cells on d3. ***F,*** Donor CD45.1^{+} and CD45.2^{+} CD4^{+} T cell counts from the spleen on d3. ***G,*** Donor CD45.1^{+} and CD45.2^{+} CD4^{+} T cell counts from the spleen on d7. ***H-I,*** On d0 recipient mice received 5x10^{6} WT B6 TCD-BM with 1x10^{6} B6 CD4^{+} T cells. ***H,*** Survival graph depicting the influence of recipient IL-33. ***I,*** Clinical scores depict the influence of recipient IL-33. Data in ***D-G*** indicate mean+/-SD, *n*=3/group. Student's *t* test (***D-F***) and one-way ANOVA (***G***). Data in ***H,*** Kaplan-Meier survival curve, ***I***, clinical scores, *n*=8/group. *, P<0.05, **, P<0.01, ***, P<0.001,

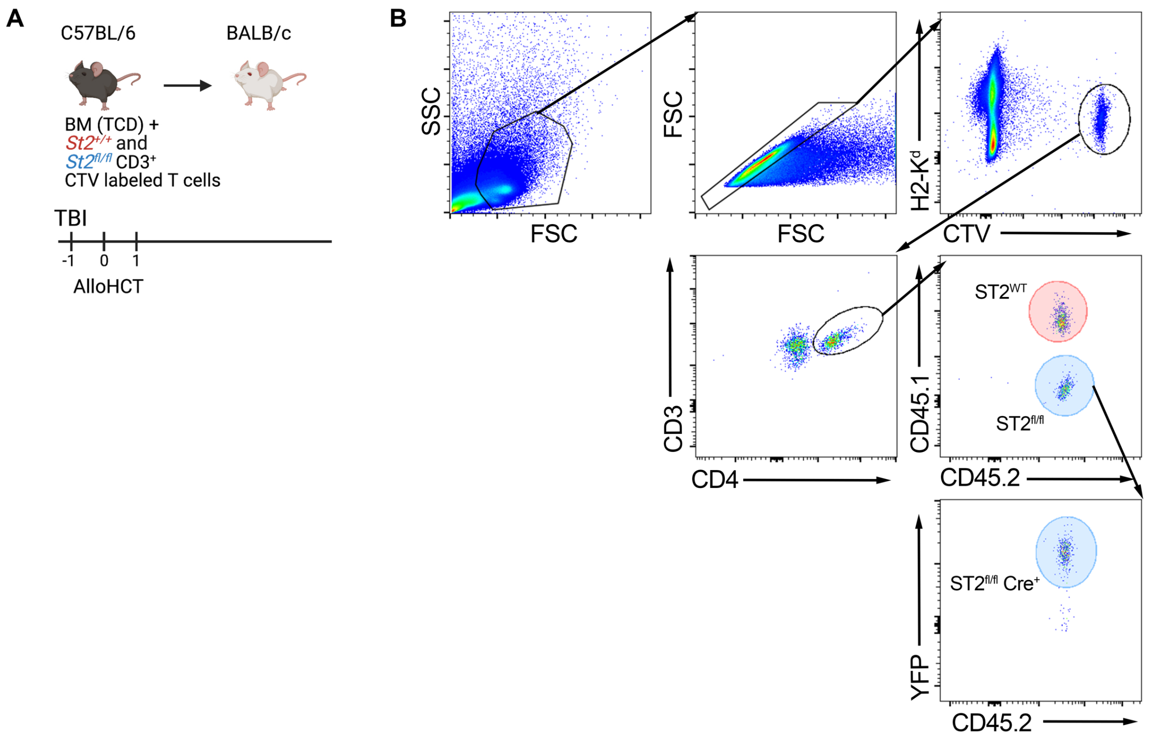

Supplemental Figure 6. Donor CD4^{+} T cells are identifiable in the spleen as early as day 1. ***A-B,*** CD3^{+} T cells from CD45.2^{+} *CD4-Cre x R26-LSL-YFP x St2$^{fl/fl}$* B6 (1x10^{6}) and *St2$^{+/+}$* CD45.1^{+} B6 (1x10^{6}) mice were labeled with CTV and adoptively transferred with 1x10^{7} WT B6 TCD-BM into lethally irradiated BALB/c recipients. ST2^{WT} (red) and ST2$^{fl/fl}$ (blue) CD4^{+} T cells from the same spleen were assessed by flow cytometry on d1. ***A,*** Schematic of mechanistic examination of the impact of ST2-mediated signaling into donor CD4^{+} T cells during GVHD initiation. ***B,*** Representative flow plot gating to identify donor CD45.1^{+}CD4^{+}YFP^{-} and CD45.2^{+}CD4^{+} YFP^{+} T cells. Data in ***A,B,*** indicate mean+/-SD, *n*=3-4/group, data are representative of 2 experiments.

Table S1. Gene signatures used in RNAseq analysis

Gene signatures	Genes included in signature
Anergy and tolerance	Cd70, Ifng, Tnfrsf8, Lgals3, Gzmb, Tbx21, Cdk4, Jun, Cd40lg, Il2rb, Cdk2, Icos, Ptger2, Il6, Il2ra, Itga1, Lat, Tnfsf14, Itch, Gata3, Il4, Cd27, Ccr4, Tnfrsf14, Dgka, Ing4, Pdcd1, Nfatc2, Tgfb1, Tnfrsf4, Mef2a, Stat3, Jak1, Tnfrsf9, Lta, Nfatc1, Cd28, Fas, Nfkb1, Tnfsf10, Jak3, Nfatc3, Stat6, Tnfsf8, Tnfrsf18, Notch1, Icam1, Dgkz, Ctla4, Foxp1, Tnfrsf10b, Prf1, Irf4, Csf2, Cblb, Fasl, Fos, Rnf128, Il2, Egr2, Foxp3, Eomes, Il7r, Il10ra, Sell, Csf1, Il10, Ccl3
T helper cell differentiation	Ifng, Tmed1, Tbx21, Socs1, Irf8, Pou2f2, Icos, Il1rl1, Il2ra, Nfatc2ip, Perp, Havcr2, Gata3, Stat4, Il4, Myb, Ccr4, Il1r2, Il18r1, Hopx, Tlr6, Rel, Nfatc2, Runx3, Irf1, Id2, Zeb1, Rora, Chd7, Zbtb7b, Tgif1, Jak1, Tnfrsf9, Nfatc1, Il18rap, Gfi1, Tnf, Nr4a1, Tnfsf11, Stat6, Maf, Stat1, Il12rb2, Socs5, Il21, Irf4, Cebpb, Runx1, Csf2, Ikzf2, Rorc, Il4ra, Il18, Pparg, Lrrc32, Fasl, Il2, Trp53inp1, Foxp3, Il1r1, Ccr3, Nr4a3, Ccl5, Ndufb7, Ndufa2, Ndufv3, Cox6b1, Ndufa3
Mitochondrial energy	Atp6v1c2, Cyc1, Ppa1, Ndufv1, Uqcrc1, Lhpp, Atp5b, Cox11, Ppa2, Ndufab1, Atp5a1, Ndufc1, Sdhd, Sdhc, Uqcrfs1, Cox5a, Ndufs7, Ndufs1, Ndufc2, Atp5g1, Atp5d, Ndufb6, Ndufb8, Ndufa10, Atp5f1, Ndufa5, Atp5c1, Atp5g3, Ndufv2, Cox7a2, Ndufb2, Ndufs2, Ndufs3, Ndufs8, Sdhb, Ndufb9, Atp5g2, Ndufb10, Ndufa8, Ndufb5, Atp5o, Atp6v1g3, Uqcrc2, Ndufa4, Atp5j, Ndufb4, Uqcr11, Ndufs5, Cox6a1, Uqcrq, Ndufs6, Atp5j2, Cox8a, Ndufa11, Bcs1l, Cox5b, Ndufs4, Sdha, Cox7b, Ndufb3, Cox4i1, Cox6c, Ndufa6, Ndufb7, Ndufa2, Ndufv3, Cox6b1, Ndufa3
Myc targets	Shmt1, Pold2, Mthfd1, Apex1, Cdc25a, Srm, Gnl3, Tpi1, Eno1, Pa2g4, Phb, Hk2, Tyms, Ccnb1, Cct5, Nolc1, Eif4a1, Psmg1, Snrpb, Ppat, Msh2, Myc, Mat2a, Cad, Cdk4, Fasn, Paics, Nap1l1, Nbn, Chek1, Ncl, Ctsc, Dkc1, Nme1, Trp53, Pcna, Csde1, E2f1, Ilk, Npm1, Ddx39b, Eif4e, Ybx3, Lta4h, Exosc8, Hnrnpa1, Odc1, Ube2c, Hnrnpa2b1, Top1, Max, Cks2, Rpl13, Srsf1, Cstb, Bcat1, Ddx10, Pten, Rpl5, Cbx3, Maz, Rpl27a, Itgb1, Rps5, Rpl19, Rpl23, Atf4, Pias2
